# Genomic analysis of a parasite invasion: Colonization of the Americas by the blood fluke *Schistosoma mansoni*

**DOI:** 10.1111/mec.16395

**Published:** 2022-02-25

**Authors:** Roy N. Platt, Winka Le Clec'h, Frédéric D. Chevalier, Marina McDew‐White, Philip T. LoVerde, Rafael Ramiro de Assis, Guilherme Oliveira, Safari Kinung'hi, Amadou Garba Djirmay, Michelle L. Steinauer, Anouk Gouvras, Muriel Rabone, Fiona Allan, Bonnie L. Webster, Joanne P. Webster, Aidan M. Emery, David Rollinson, Timothy J. C. Anderson

**Affiliations:** ^1^ Texas Biomedical Research Institute San Antonio Texas USA; ^2^ University of Texas Health Science Center San Antonio Texas USA; ^3^ Centro de Pesquisas René Rachou—Fiocruz/MG Belo Horizonte Brazil; ^4^ Instituto Tecnológico Vale Belém Brazil; ^5^ National Institute for Medical Research Mwanza Tanzania; ^6^ Réseau International Schistosomiases Environnemental Aménagement et Lutte (RISEAL) Niamey Niger; ^7^ Western University of Heath Sciences Corvallis Oregon USA; ^8^ Natural History Museum London UK; ^9^ Department of Pathobiology and Population Sciences Royal Veterinary College, Centre for Emerging, Endemic and Exotic Diseases University of London Hertfordshire UK; ^10^ London Centre for Neglected Tropical Disease Research, Imperial College London School of Public Health London UK

**Keywords:** Africa, Brazil, codispersal, exome, human parasite, migration

## Abstract

*Schistosoma mansoni*, a snail‐borne, blood fluke that infects humans, was introduced into the Americas from Africa during the Trans‐Atlantic slave trade. As this parasite shows strong specificity to the snail intermediate host, we expected that adaptation to South American *Biomphalaria* spp. snails would result in population bottlenecks and strong signatures of selection. We scored 475,081 single nucleotide variants in 143 *S. mansoni* from the Americas (Brazil, Guadeloupe and Puerto Rico) and Africa (Cameroon, Niger, Senegal, Tanzania, and Uganda), and used these data to ask: (i) Was there a population bottleneck during colonization? (ii) Can we identify signatures of selection associated with colonization? (iii) What were the source populations for colonizing parasites? We found a 2.4‐ to 2.9‐fold reduction in diversity and much slower decay in linkage disequilibrium (LD) in parasites from East to West Africa. However, we observed similar nuclear diversity and LD in West Africa and Brazil, suggesting no strong bottlenecks and limited barriers to colonization. We identified five genome regions showing selection in the Americas, compared with three in West Africa and none in East Africa, which we speculate may reflect adaptation during colonization. Finally, we infer that unsampled populations from central African regions between Benin and Angola, with contributions from Niger, are probably the major source(s) for Brazilian *S. mansoni*. The absence of a bottleneck suggests that this is a rare case of a serendipitous invasion, where *S. mansoni* parasites were pre‐adapted to the Americas and able to establish with relative ease.

## 1 INTRODUCTION

Genomic characterization of parasites and pathogens is increasingly being used as an aid to traditional epidemiological methods in reconstructing transmission patterns (de Oliveira et al., 2020; Nadeau et al., 2021). On a longer timescale, genomic data can be used to understand biological invasions of pathogens into new continents, just as these methods are used for investigating biological invasions in free‐living organisms (Rius et al., 2015; Sherpa & Després, 2021). Such methods can determine the colonization route, source population, number of colonization events, whether diversity is reduced during colonization and evidence for adaptation in colonizing populations. Examining the consequences of historical invasions can inform our understanding of extant invasions.

The Trans‐Atlantic slave trade lasted from 1502 to 1888 (Bergad, 2007). During this time, more than 12 million people were trafficked from Africa to slave ports in the Americas, representing one of the largest forced migration events in human history (Eltis, 2001). Along with the human cargo, a number of human pathogens were introduced into the Americas. For example, parvovirus B19 (*Primate erythroparvovirus 1*) and *Hepatitis B virus* were successfully introduced into the Americas, leading to large‐scale outbreaks (Guzmán‐Solís et al., 2021). Today viable populations of pathogens including herpes simplex virus 2 (*Human alphaherpesvirus 2*; Forni et al., 2020), *Yellow fever virus* (Bryant et al., 2007), the parasitic nematode *Wuchereria bancrofti* (Small et al., 2019), among others (Steverding, 2020), are all a direct result of introductions during the Trans‐Atlantic slave trade. In some cases, the genetic signatures of the introduction are still visible. For example, genetic diversity in South American *Leishmania chagasi* populations is halved and the effective population size (*N*_e_) is reduced from 43.6 million to 15.5 thousand compared to source populations in Africa (Leblois et al., 2011; Schwabl et al., 2021). Here, we focus on another successful invasion by a human‐parasitic trematode, *Schistosoma mansoni*.

*S. mansoni* is distributed from Oman, through sub‐Saharan Africa, to the Caribbean and countries along the eastern coast of South America. Phylogenetic evidence indicates that *S. mansoni* in West Africa and the Americas are closely related (Crellen et al., 2016; Desprès et al., 1993; Fletcher et al., 1981; Morgan et al., 2005; Webster et al., 2013) and these observations, along with demographic reconstructions (Crellen et al., 2016), indicate a recent origin of *S. mansoni* in the Americas. As a result, there is strong evidence that *S. mansoni* comigrated to the Americas during the forced, human migrations of the Trans‐Atlantic slave trade (Files, 1951). Furthermore, reduced diversity in mitochondrial haplotypes (Desprès et al., 1993; Fletcher et al., 1981; Morgan et al., 2005; Webster et al., 2013) in South American *S. mansoni* suggests the presence of a bottleneck during parasite establishment.

Our central goal here was to use parasite genomic data to investigate this human‐mediated, biological invasion and the impacts of a relatively recent, transcontinental, migration event. Parasites in the genus *Schistosoma* have a complex life cycle involving human definitive hosts and aquatic snail intermediate hosts (reviewed in Anderson & Enabulele, 2021). Eggs are expelled in human faeces (*S. mansoni* and *S. japonicum*) or urine (*S. haematobium*). Larvae (miracidia) hatch in fresh water and infect receptive snails. Inside the snail host, the schistosomes reproduce asexually, and second‐stage larvae (cercariae) are released back into the water where they infect humans, mature into adult worms and restart their life cycle. *S. mansoni* is diploid, with a well‐characterized 363‐Mb genome (Berriman et al., 2009; International Helminth Genomes Consortium, 2019; Protasio et al., 2012), ZW sex determination, obligate sexual reproduction of adult worms, and a relatively long lifespan (5–10 years) (Fulford et al., 1995).

The distribution of the intermediate snail host is a major driver of schistosome distribution. *S. mansoni* shows strong specificity for species and even strains of snails in the genus *Biomphalaria* (Webster & Woolhouse, 1998). However, *Biomphalaria pfeifferi*, *B. sudanica* and *B. alexandrina* are the primary intermediate hosts in Africa (DeJong et al., 2001), while *B. glabrata*, *B. tenagophila* and *B. straminea* are the known snail hosts in South America (Vidigal et al., 2000). *S. mansoni* infections can impact the reproductive viability of their snail hosts, and there are strong co‐evolutionary interactions driving resistance to infection in snails and for infectivity in parasites (Davies et al., 2001; Theron et al., 2014; Webster et al., 2004). Several schistosome resistance genes have been localized within the snail genome (Tennessen et al., 2015, 2020) and polymorphic loci in snails and parasites are thought to determine compatibility (Mitta et al., 2017; Webster & Woolhouse, 1998; Woolhouse & Webster, 2000). Based on these observations, we hypothesize that the adaptation to novel *Biomphalaria* spp. hosts would place strong selective pressures on *S. mansoni* as it became established in the Americas.

Adult schistosomes live in the blood vessels, making them difficult to sample. Genome and exome sequencing of schistosomes is now possible using whole genome amplification of miracidia larvae isolated from faeces or urine (Doyle et al., 2019; Le Clec'h et al., 2018; Shortt et al., 2017), and several genome‐scale population analyses have recently been published (Berger et al., 2021; Platt et al., 2019; Shortt et al., 2017). Our goal here was to address the following questions with the available sequence data from both Africa (Niger, Senegal, Uganda, Tanzania) and the Americas (Caribbean, Brazil): (i) Are the genomic data consistent with a West African origin of colonizing schistosome populations? (ii) Is there evidence for genetic bottlenecks during colonization? (iii) Are there genomic signatures suggesting adaptation of colonizing parasites to the Americas? (iv) Can we determine the source country, or countries, for parasite populations in the Americas?

## 2 MATERIALS AND METHODS

### 2.1 Data and sample information

**FIGURE 1:**
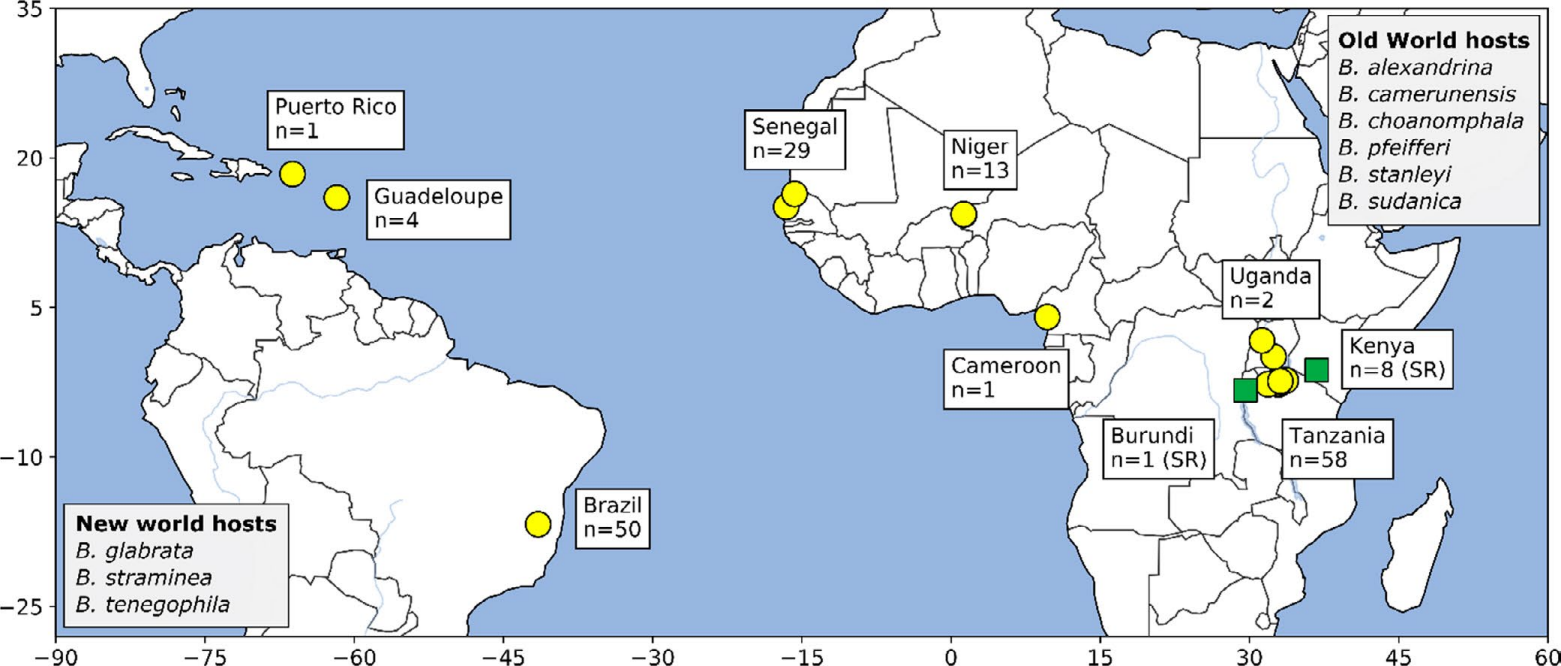
Sampling localities. Location and number of samples for *Schistosoma mansoni* (yellow circles) and *S. rodhaini* (green squares) samples included in this study. Members of the genus *Biomphalaria* are the predominant intermediate hosts with specific distributions of the different species involved in transmission varying across Africa and South America. Intermediate snail vectors are listed in greyed boxes

We examined published exomic and genomic data from 178 individual *Schistosoma* samples/isolates, from multiple geographical locations, available from three studies (Berriman et al., 2009; Chevalier et al., 2019; Crellen et al., 2016). Exome data are from Chevalier et al. (2016) and Chevalier et al. (2019). These data were generated from individual larval miracidia hatched from *Schistosoma mansoni* eggs and preserved on Flinders Technology Associates^®^ (FTA) cards. Exome libraries were generated via whole genome amplification followed by targeted capture of the exome (Le Clec'h et al., 2018). This method specifically targets 95% (14.81 Mb) of the exome with 2× tiled probes. The whole genome sequence data came from adult worms cultured through laboratory rodents and snails for two or more generations before whole genome library preparation and sequencing (Berriman et al., 2009; Crellen et al., 2016; International Helminth Genomes Consortium, 2019). Sample origins are shown in Figure 1. Detailed metadata are available for each sample in Table S1 including country of origin, species identification and NCBI Short Read Archive.

### 2.2 Computational environment

We used conda version 4.8.3 to manage computational, virtual environments for all analyses. Sequence read filtering through genotyping steps were documented in a snakemake version 5.18.1 (Köster & Rahmann, 2012) workflow and all other analyses were performed in a series of jupyter version 1.0.0 notebooks. The code for this project, including shell scripts, snakemake workflows notebooks, and environmental yaml files are available at https://github.com/nealplatt/sch_man_nwinvasion/releases/tag/v0.2 (last accessed October 21, 2021) and accessioned at https://doi.org/10.5281/zenodo.5590460 (last accessed October 21, 2021).

### 2.3 Genotyping

Paired‐end reads were quality filtered with trimmomatic version 0.39 (Bolger et al., 2014) with the following parameters: “LEADING:10TRAILING:10 SLIDINGWINDOW:4:15MINLEN:36.” Filtered reads were mapped to the *S. mansoni* genome (GenBank Assembly accession: GCA_000237925.3) using bwa version 0.7.17‐r1188 (Li & Durbin, 2010). We allowed up to 15 mismatches per 100‐bp read (‐n 15) to account for divergence between *S. mansoni* and *S. rodhaini*. Mapped single and paired reads were merged into a single file and all optical/PCR (polymerase chain reaction) duplicates were removed with gatk version 4.1.2.0’s (McKenna et al., 2010) MarkDuplicates. Single nucleotide variants (SNVs) were called with HaplotypeCaller and GenotypeGVCFs on a contig‐by‐contig basis, combined into a gvcf per individual, and finally merged into a single gvcf for the entire data set. We used high‐quality, SNV data from Le Clec’h et al. (2021) as a training data set for variant recalibration and scored SNV quality using “‐an SOR ‐an MQ ‐an MQRankSum ‐an ReadPosRankSum.” Sensitivity Tranches (‐‐truth‐sensitivity‐tranche) were set at 100, 99.5, 99, 97.5, 95 and 90. We recalibrated SNVs using the 97.5 sensitivity tranche and filtered low‐confidence sites with the following set of filters (‐‐filter‐expression): QD < 2.0, MQ < 30.0, FS > 60.0, SOR > 3.0, MQRankSum < −12.5 and ReadPosRankSum < −8.0." All genotyping steps from read filtering through variant recalibration were contained within a single snakemake version 5.18.1 (Köster & Rahmann, 2018) script.

We used vcftools version 0.1.16 (Danecek et al., 2011) for additional rounds of filtering. First, we removed low‐quality sites with quality score <25, read depth <12 and nonbiallelic sites. Second, we removed sites and individuals with a genotyping rate <50%. Third, we removed all sites that were on unresolved haplotigs by retaining only those SNVs that were on one of seven autosomal scaffolds (GenBank Nucleotide accessions: HE601624.2‐30.2), the sex‐linked ZW scaffold (HE601631.2) or the mitochondria (HE601612.2). Finally, for analyses requiring unlinked SNVs, we filtered linked sites within 250‐kb windows using plink version 1.90b4 (Purcell et al., 2007) with the following parameters: “—indep‐pairwise 250 kb 1 0.20.”

### 2.4 Summary statistics

We quantified read depth per probed‐exome region with mosdepth version 0.2.5 (Pedersen & Quinlan, 2018) and calculated genome‐wide summary statistics for each population, including *F*_3_, *F*_ST_, Tajima's *D*, *π*, and the Watterson estimator (Θ) with scikit‐allele version 1.2.1 (Miles et al., 2019). We examined genome regions that were targeted by the Le Clec'h et al. (2018) probe set for these calculations; nontarget regions (i.e., nonexomic) were ignored because most samples lacked information from these regions. *F*_ST_ between populations was calculated from the average Weir–Cockerham *F*_ST_ (Weir & Cockerham, 1984) in windows of 100 SNVs. Effective population size (*N*_e_) was estimated from Θ and the mutation rate (*μ* = 8.1e‐9 per base per generation; Crellen et al., 2016) with:

$$N_{e}=\frac{\Theta }{4\mu }.$$

We examined linkage disequilibrium (LD) within each population by calculating *r* ^2^ (‐‐r2) values with plink version 1.90b6.18 (Purcell et al., 2007). We excluded invariant sites from the analyses. Intra‐autosomal, pairwise comparisons between SNVs within 1 Mb of one another were allowed by setting the following parameters: “‐‐ldwindow 1000000”, “‐‐ld‐window‐kb 1000” and “‐‐ld‐window‐r2 to 0.0.” *r* ^2^ values were then binned into 500‐bp windows and averaged for each population using the R version 3.6.1 stats.bin function in the fields version 11.6 (Nychka et al., 2017) library. We used local regression to smooth the binned *r* ^2^ values with the loessMod function in the base R version 3.6.1 package and a span size of 0.5.

We used a Pearson Mantel test to examine correlation between genetic and physical distance. Since we did not have exact collection coordinates from whole genome samples, or they were laboratory‐derived, we excluded them from the analyses and instead focused only on the *S. mansoni* exome samples. We calculated pairwise p‐distances with vcf2dis (https://github.com/BGI‐shenzhen/VCF2Dis; commit: b7684d3, accessed February 13, 2021) and physical distances between samples the Python haversine 2.3.0 module. Finally, we used the mantel() function in the scikit‐bio 0.2.1 Python library to conduct a Pearson Mantel test that included 1000 permutations.

### 2.5 Population structure and admixture

We examined population substructure using principal components analysis (PCA) and admixture with unlinked autosomal SNVs (described above). Two PCAs were calculated in plink version 1.90b6.18, with and without the *S. rodhaini* samples. Population ancestry was estimated with admixture version 1.3.0 (Alexander et al., 2009). We examined between *k* = 1 and *k* = 20 populations and used the vross‐validation scores and the Evanno et al. (2005) method to determine a range of viable *k* value. Q estimates were used as a proxy for ancestry fractions.

We examined *D* (Patterson et al., 2012), *D*_3_ (Hahn & Hibbins, 2019) and *F*_3_ (Patterson et al., 2012) to identify gene flow between *S. rodhaini* and *S. mansoni* with emphasis on the Tanzanian *S. mansoni* population since it and the *S. rodhaini* samples are from East Africa. For *D*_3_, we calculated the mean‐pairwise (Euclidean) distances between populations using scikit‐allele’s allel.pairwise_distance() function. To determine significance, we used 1000 block bootstrap replicates of 1000 SNV blocks. We calculated the average *F*_3_ across the genome in blocks of 100 variants. Here we ran multiple tests that included some combination of an African *S. mansoni* population (Niger, Senegal and Tanzania) as the test group and Brazilian *S. mansoni* and *S. rodhaini* as the potential source populations. *D*, or the ABBA‐BABBA statistic, was averaged over blocks of 1000 variants assuming a phylogeny of (((*a*, Tanzania), *S. rodhaini*), *S. margrebowiei*), where the *a* population was either Brazil, Niger or Senegal. *D*, *D*_3_ and *F*_3_ values were calculated using scikit‐allel.

### 2.6 Phylogenetics

We used three different phylogenetic methods to visualize relationships among sampled schistosomes: a mitochondrial haplotype network, a coalescent‐based species tree and a phylogenetic network.

#### 2.6.1 Mitochondrial haplotype network

We extracted mitochondrial SNVs from all *S. mansoni* individuals with vcftools and converted the subsequent VCF file to Nexus format with vcf2phylip version 2.0 (Ortiz, 2019). A median joining network (*ε* = 0) was created from the mitochondrial haplotypes with popart version 1.7 (Leigh & Bryant, 2015).

#### 2.6.2 Coalescent‐based species tree

We generated a coalescent‐based species tree with svdquartets (Chifman & Kubatko, 2015) packaged in paup* version 4.0.a.build166 (Swofford, 2003). We examined parsimony‐informative, autosomal SNVs by removing private alleles (singleton and doubletons). All samples were assigned to a population based on their country of origin (examples include Niger, Puerto Rico, Brazil, Cameroon, etc.) except for the laboratory‐derived, Caribbean samples. Each of these samples was considered to represent an individual population given their histories of extensive laboratory passage (exclusing Guadeloupe1, Guadeloupe2, Puerto Rico). We randomly evaluated 100,000 random quartets and bootstrapped the quartet tree with 1000 standard replicates. The tree was rooted on the single *S. margrebowiei* individual.

#### 2.6.3 Phylogenetic network

We used a phylogenetic network to visualize and quantify migration among schistosome populations. We only included *S. mansoni* populations with more than four individuals, which excluded all whole genome samples from this analysis including those from the Caribbean and the *S. rodhaini* samples. We used autosomal SNVs after filtering linked sites in 250‐kb blocks with plink version 1.90b6.18 and then used treemix version 1.12 (Pickrell & Pritchard, 2012) to generate the phylogenetic network. This analysis used a covariance matrix generated from blocks of 500 SNVs without sample‐size correction (“‐‐noss”) and the number of migration events was limited to 3.

### 2.7 Selection

We scanned the genome to identify regions under selection using haplotype (h‐scan version 1.3; Schlamp et al., 2016), allele frequency (sweepfinder2 version 2.1; DeGiorgio et al., 2016) and PCA‐based (pcadapt version 4.3.3; Luu et al., 2017) methods. In addition, to avoid false positives, we used msprime version 0.7.4 (Kelleher et al., 2016) to conduct simulations to estimate the range of values expected under neutrality from h‐scan and sweepfinder2.

#### 2.7.1 Neutral simulations

We used msprime version 0.7.4 (Kelleher et al., 2016) to simulate a set of neutrally evolving SNVs along a single chromosome for each population and then used the simulated data with h‐scan and sweepfinder2 to define the range of values expected in the absence of selection. For these simulations we used a mutation rate (*μ* = 8.1e‐9 per base per generation; Crellen et al., 2016) and recombination rate (3.4e‐8 per base per generation; Criscione et al., 2009) from previous work on *S. mansoni*. Population‐specific estimates of *N*_e_ are described above. The simulated chromosome length was set to 88.9 Mb which is equal to chromosome 1 (HE601624.2) in the *S. mansoni* assembly. The number of chromosomes sampled per msprime run was equal to the number of samples available from each population. We used these parameters to perform 342 simulations for each population, roughly equivalent to 100 genomes of simulated data. Next, we downsampled the SNVs along the entire simulated chromosome so that they were comparable with our targeted sequencing approach (i.e., only SNVs from “exomic” regions). Since the simulated chromosomes were the same size as chromosome 1, we transposed the chr1 annotation onto the simulated chromosome and extracted only those SNVs occurring in regions accessible by our biotinylated probes with vcftools. The simulated data were run through h‐scan and sweepfinder2 in parallel with the actual SNV data to establish a range of neutral values for each test (described above).

#### 2.7.2 h‐scan

This method measures the length of homozygous haplotypes to identify regions under selection. Strong selection will drive adaptive alleles linked SNVs to high frequency and reduce homozygosity in the surrounding region. For each population, we converted autosomal SNVs from VCF to h‐scan format using vcf2hscan.py script from vcf2phylip version 2.0 (Ortiz, 2019) and ran h‐scan on each chromosome. To avoid calculating *H* across large distances without variants we set a maximum gap length (‐g) of 10 kb. *H* values were smoothed (*H*_smoothed_) by median filtering values in 201 SNV windows (step size =1) using the medfilt function in scipy version 1.5.2 (Virtanen et al., 2020) for visualization purposes.

#### 2.7.3 sweepfinder2

This method uses deviations in allele frequency from a neutral expectation to estimate positive selection while accounting for the possibility of background selection via a likelihood ratio (LR) test (DeGiorgio et al., 2016). Empirical site‐frequency spectra were calculated for each population, and within each population LR was estimated along each autosome individually. We examined grid points (“g”), or window sizes, of 1, 5, 10 and 20 kb to accommodate possible gaps caused by exome data. These options had minimal impact on the results. Downstream analyses are reported on the runs with “g” =1 kb.

#### 2.7.4 pcadapt

We used the R version 4.0.5 package pcadapt version 4.3.3 (Luu et al., 2017) to identify highly differentiated loci among populations via variants associated with population structure as identified by PCA. We only included samples from Brazil, Niger and Senegal since our primary goal was to identify variants involved in adaptation to the Americas. Rare variants (MAF < 5%) were excluded with vcftools version 0.1.16. We identified the appropriate number of principal components from the data by running an initial pcadapt run with 20 populations (*K* = 20) and LD filtered variants (LD.clumping = list(size =100, thr =0.2)). The major break in the subsequent scree plot was used as the optimal *K* choice. We used a second pcadapt run with the optimal *K* and the same LD filtering parameters as the initial run to assign *p* values to each site. Finally, we adjusted *p* values for multiple tests with Bonferroni correction and an *α* = .05 to identify SNV outliers associated with population differentiation.

#### 2.7.5 Identifying regions of selection

We identified regions potentially under positive selection using a three‐step process. First, we identified SNVs whose h‐scan and sweepfinder2 values were in the 99th percentile of and greater than the neutral thresholds established with msprime. These were SNVs with the strongest signal of selection. Then, we expanded from the SNV to a broader region by merging all variants within 333,333 bp whose h‐scan or sweepfinder2 values were greater than the neutral thresholds. Finally, we looked for pcadapt outliers in each region. These regions are referred to as “putative selected regions” or “putative regions of selection.” Gene names and functions were taken from UniProtKB (release 2020_06) or HHsearch (Steinegger et al., 2019) annotations from Le Clec'h et al. (2021). The entire process is summarized in Figure S1.

## 3 RESULTS

### 3.1 Summary of sequence data

After genotyping and filtering, we removed 25 of the 178 samples with low numbers of reads, poor coverage or low genotyping rates. The final data set included 135 *Schistosoma mansoni* (exome), eight *S. mansoni* (genome), eight *S. rodhaini* (exome), one *S. rodhaini* (genome) and one *S. margrebowiei* (genome). We genotyped 1,823,890 sites, which was reduced to 475,081 autosomal and 815 mitochondrial variants after quality filtering: one variant per 641 bases. Further, because we required that any site be genotyped in >50% of the samples and only 11% (18 of 153) of the samples were from whole genome data, all of the variants outside of the exome were removed. The final data set comprised 153 samples with mean read depths of 520.7× (range: 251.4–998.2×) and 66.0× (range: 14.8–726.2×) at mitochondrial and autosomal loci. Location and sequence coverage statistics for all samples in the final data set are listed in Table S1.

### 3.2 Summary statistics

Autosomal and mitochondrial summary statistics for *π*, *H*, Tajima's *D*, Θ and *N*_e_ are shown in Table 1. *π*, Θ and *N*_e_ are similar between the West African and Brazilian *S. mansoni* populations but are two to three times lower than observed in Tanzanian *S. mansoni*. Tajima's *D* values ranged from slightly positive to negative (Tajima's *D* = −1.417 to 0.034) in *S. mansoni* (Table 1; Table S2). All the African populations show negative Tajima's *D*, consistent with natural selection or population expansion. However, the Brazilian Tajima's *D* is positive, which is inconsistent with a bottleneck during colonization of South America. Mitochondrial diversity was quantified with *π* and haplotype diversity (*H*). *H* in all populations is very high (>0.978) indicating that all mitochondrial haplotypes are unique. Mitochondrial *π* follows the same pattern as autosomal *π* with the exception that the mitochondrial *π* in Brazil is lower than expected when compared to measures in Niger and Senegal. *F*_ST_ values between *S. mansoni* populations are shown in Table 2 and were highest in pairwise comparisons that included the Tanzanian population. Mantel tests showed significant signs of isolation‐by‐distance within Africa (*r* = .64, *p* = .001) and between African and Brazilian (*r* = .77, *p* = .001) *S. mansoni* samples.

**TABLE 1:** Whole genome summary statistics for *Schistosoma mansoni* populations and *S. rodhaini*

	*n*	*π*	*π* (mito)	*H* (mito)	Tajima's *D*	Θ	*N*_e_	*π* (PRS)	Tajima's *D* (PRS)
*S. rodhaini*	9^a^	5.61E‐04	Na^b^	Na^b^	0.479	4.29E‐04	13,226	Na^c^	Na^c^
Sm (Brazil)	45	6.93E‐04	2.10E‐03	0.984	0.034	5.84E‐04	18,032	2.22E‐04	−1.218
Sm (Niger)	10	6.00E‐04	5.89E‐03	0.978	−0.579	6.07E‐04	18,737	2.46E‐04	−1.018
Sm (Senegal)	25	4.97E‐04	4.72E‐03	0.997	−1.417	−1.375	21,992	1.75E‐04	−1.816
Sm (Tanzania)	55	1.45E‐03	7.25E‐03	1.0	−0.729	−0.739	51,508	Na^c^	Na^c^

Note

"*n*"—number of samples; "*π*"—nucleotide diversity; "*H"*—haplotype diversity; "Θ"—Watterson estimator; "*N*_e_"—effective population size.

Abbreviations: PRS, putative region of selection; Sm, *Schistosoma mansoni*.

^a^ Eight of nine *S. rodhaini* samples came from a single laboratory population: population statistics are probably biased.

^b^ No *S. rodhaini* reads mapped to the *S. mansoni* mitochondria.

^c^ Not calculated.

**TABLE 2:** *F*_ST_ between *Schistosoma* species and populations

Pop1	Pop2	*F*_ST_	*SE*
*S. rodhaini*	Sm (Caribbean)	0.929	0.0012
*S. rodhaini*	Sm (Tanzania)	0.844	0.0016
*S. rodhaini*	Sm (Senegal)	0.937	0.0013
*S. rodhaini*	Sm (Niger)	0.931	0.0011
*S. rodhaini*	Sm (Brazil)	0.919	0.0013
Sm (Caribbean)	Sm (Tanzania)	0.279	0.0036
Sm (Caribbean)	Sm (Senegal)	0.323	0.0085
Sm (Caribbean)	Sm (Niger)	0.236	0.0071
Sm (Caribbean)	Sm (Brazil)	0.154	0.0067
Sm (Tanzania)	Sm (Senegal)	0.416	0.0032
Sm (Tanzania)	Sm (Niger)	0.348	0.0031
Sm (Tanzania)	Sm (Brazil)	0.379	0.0034
Sm (Senegal)	Sm (Niger)	0.135	0.0042
Sm (Senegal)	Sm (Brazil)	0.235	0.0047
Sm (Niger)	Sm (Brazil)	0.152	0.0036

Abbreviation: Sm, *Schistosoma mansoni*.

Diversity and *N*_e_ values were lower in *S. rodhaini* than all *S. mansoni* populations but should be interpreted with caution. Eight of the nine *S. rodhaini* samples are from the same laboratory‐maintained population. *F*_ST_ between *S. rodhaini* and individual *S. mansoni* populations ranged from 0.844 (*S. rodhaini* vs. *S. mansoni* Tanzania) to 0.937 (*S. rodhaini* vs. *S. mansoni* Senegal).

**FIGURE 2:**
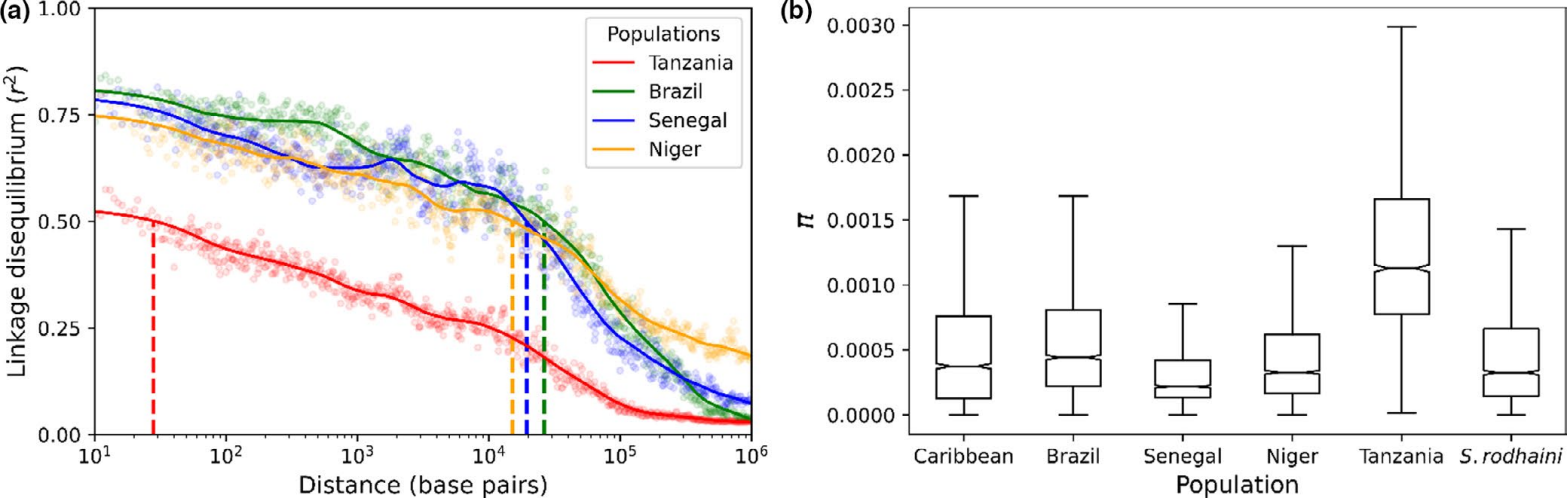
Linkage disequilibrium (LD) decay and diversity within populations—(a) LD between single nucleotide variants was quantified with *r* ^2^ values for each population. Mean *r* ^2^ values were taken in 500‐bp windows and loess smoothed. Vertical dotted lines indicate the distance where *r* ^2^ = .5 for each population. LD decayed to *r* ^2^ = .5 in 28 bp (Tanzania), 15,150 bp (Niger), 19,318 bp (Senegal) and 26,196 bp (Brazil). (b) Nucleotide diversity (*π*) varied between *Schistosoma mansoni* populations with the highest levels of diversity occurring in East Africa (Tanzania). *π* was measured in 100‐kb windows across the autosomal chromosomes. Outliers are not shown

Figure 2 shows LD decay (binned and smoothed) from pairwise *r* ^2^ values. LD decays to *r* ^2^ ≤ .2 within 500 kb for all populations. LD was weakest in the Tanzania population with *r* ^2^ decaying to .5 in 28 bp. LD decay in the other three populations (Senegal, Niger and Brazil) was relatively consistent with LD decaying by half (*r* ^2^ = .5) between 15,150 bp (Niger) and 26,196 bp (Brazil). Both Senegal and Niger show high levels of LD even between SNVs on different chromosomes: this results from lower sample size (Senegal, *n* = 25; Niger, *n* = 10) in these two populations.

### 3.3 Admixture with *S. rodhaini*

We asked whether hybridization with *S. rodhaini*, a closely related schistosome infecting rodents, might contribute to the high genetic diversity observed in East Africa vs. West Africa and South American *S. mansoni*. To investigate this, we used three statistics (*D*, *D*_3_ and *F*_3_) to test for admixture between *S. mansoni* and *S. rodhaini*, with particular emphasis on the Tanzanian populations of *S. mansoni*. Each of these statistics attempts to identify the presence of admixture in different ways. *D* and *D*_3_ values ≠0 indicate admixture with positive and negative values determining the direction of introgression. *F*_3_ values < 0 indicate admixture between the two source populations. None of the three statistics, or any of the population combinations, returned values containing significant signals of admixture (Table 3). These results suggest that hybridization/introgression between *S. mansoni* and *S. rodhaini* may make no detectable contribution to elevated diversity in East Africa.

**TABLE 3:** Admixture statistics

Comparison	Test Stat	*SE*	*Z*
*D* (Patterson et al., 2012)			
(((Br, Tz)Sr), Smr)	−0.033	0.0404	−0.807
(((Ni, Tz)Sr), Smr)	0.006	0.0437	0.129
(((Se, Tz)Sr), Smr)	0.051	0.0473	1.070
*F*_3_ (Patterson et al., 2012)			
Br: Tz, Sr	0.764	0.0207	36.910
Ni: Tz, Sr	0.993	0.0299	33.214
Se: Tz, Sr	1.265	0.0461	27.428
*D*_3_ (Hahn and Hibbens 2019)			
(Br, Tz, Sr)	−0.0047	0.00036	0.418
(Ni, Tz, Sr)	−0.0046	0.00034	0.419
(Se, Tz, Sr)	−0.0054	0.00048	0.353
(Cm, Tz, Sr)	0.0005	0.00066	−0.022
(Cr, Tz, Sr)	−0.0048	0.00029	0.517
(Ug, Tz, Sr)	0.0034	0.00062	−0.174

Note

"*Z*"—*Z*‐score of the mean from 0.

Population abbreviations: Br, Brazil; Cm, Cameroon; Cr, Caribbean; Ni, Niger; Se, Senegal; Smr, *Schistosoma margrebowiei*; Sr, *Schistosoma rodhaini*; Tz, Tanzania; Ug, Uganda.

### 3.4 Population structure

**FIGURE 3:**
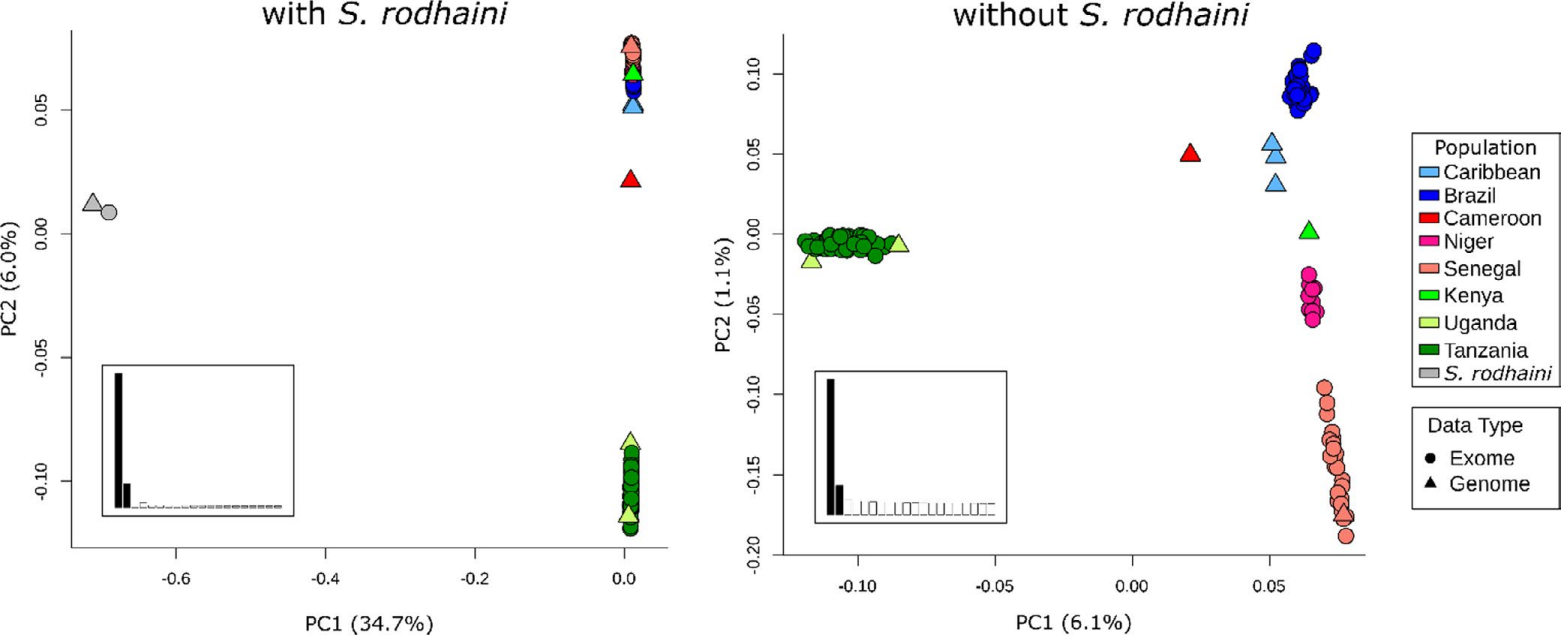
A principal component analysis (PCA) of unlinked autosomal single nucleotide variants. The PCA plot that included *Schistosoma mansoni* and *S. rodhaini* (left) clearly shows a large distinction between the two species with some variation within *S. mansoni* along PC2. A PCA with only *S. mansoni* (right) differentiates East African *S. mansoni* along PC1. The remaining *S. mansoni* samples fall along a continuum on PC2 that goes from samples in West Africa and transitions to the Americas. Inset bar charts represent the percentage variation explained along the first 20 PCs. Only PC1 and PC2 were examined (shaded)

**FIGURE 4:**
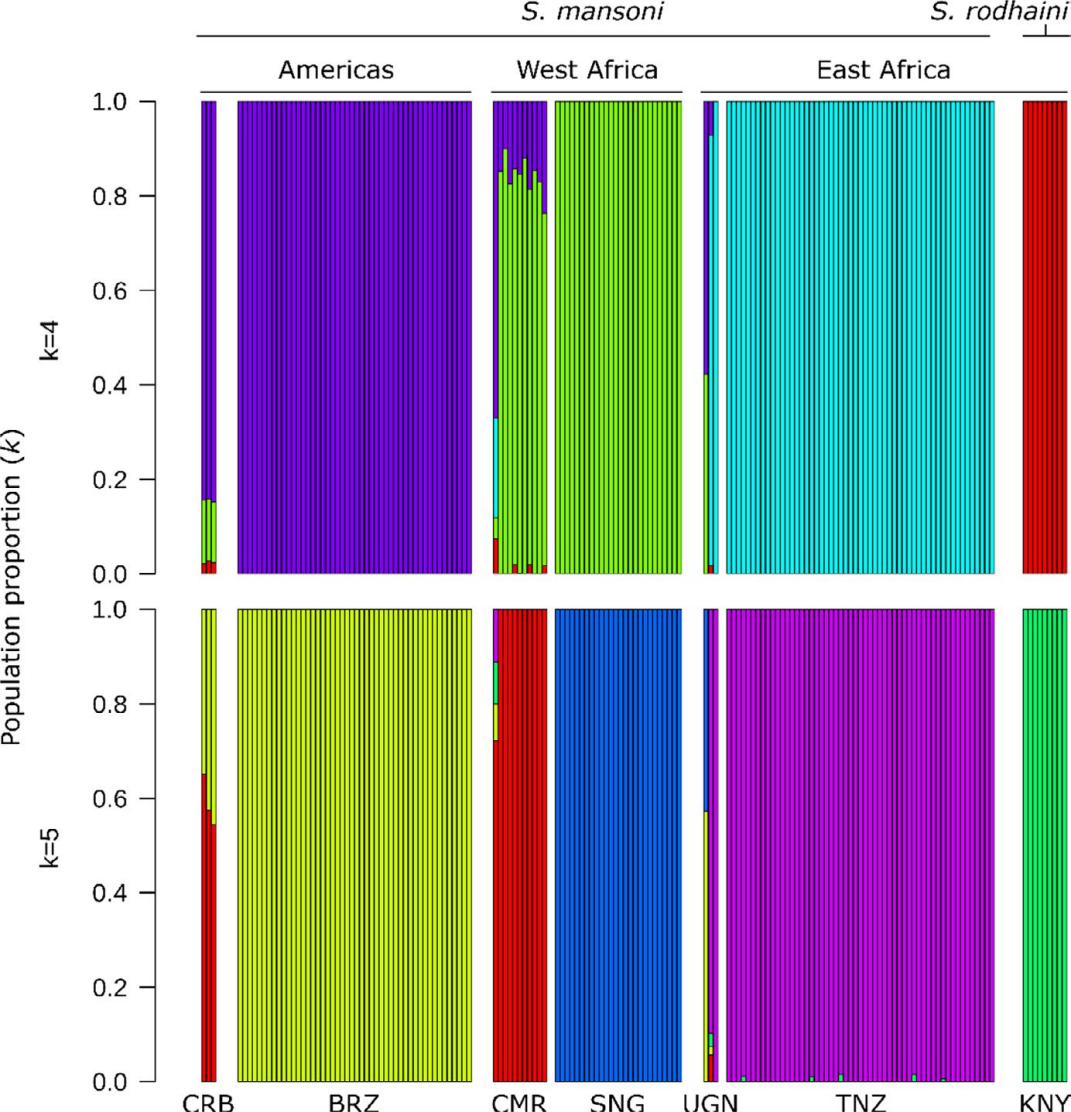
Population structure in *Schistosoma mansoni*. admixture analyses with *k* = 4 and *k* = 5 populations identified clear distinctions between each of the major sampling localities (the Americas, West Africa, East Africa and *S. rodhaini*). The population components in each of the whole genome samples from Uganda (UGN), Kenya (KNY), Cameroon (CMR) and the Caribbean (CRB) were more heterogeneous than samples with exome data. Cameroonian and Nigerien samples contain moderate proportions of the Brazilian population component. BRN, Burundi; BRZ, Brazil; NGR, Niger; SNG, Senegal; TNZ, Tanzania

We examined population structure using PCA and admixture with 38,197 unlinked autosomal SNVs. Two PCAs were generated, with and without the *S. rodhaini* outgroup (see Figure 4). The two species were differentiated along PC1 (34.7% variance) when *S. rodhaini* was included (Figure 3a). *S. mansoni* samples cluster into geographically defined groups when *S. rodhaini* is excluded (Figure 3b). East African samples were distinct from all other *S. mansoni* samples, except for one whole genome sample collected in Kenya (see below). Samples from the Americas, including those from the Caribbean and Brazil, showed a closer relationship with Cameroon and Nigerien samples than those from Senegal.

We used admixture to assign individuals to one of *k* populations, where *k* is between 1 and 20 (Figure 4). Cross‐validation scores (Evanno et al., 2005) were minimized when *k* was 4 or 5. Both *k* = 4 and 5 split *S. mansoni* samples into geographically defined populations with two major differences. First, *k* = 4 showed that the allelic component primarily associated with Brazil was found at moderate levels in Cameroon and Nigerien individuals. Second *k* = 5 split the West African samples into a Senegalese and a Cameroonian + Nigerien population.

As observed in the PCA, the Kenyan, whole‐genome sample contained a large portion of alleles associated with samples from the Americas (~40%–60%) in the admixture analysis. Crellen et al. (2016) recovered similar results and hypothesized that the Kenyan sample may be reflecting human‐trafficking routes between Portuguese and Arab slave traders out of the port of Mombasa. Given that only a single sample is available from this region, and contamination with South American strains during laboratory passage is a possible alternative explanation, we chose to remove the Kenyan sample from downstream analyses.

### 3.5 Phylogenetics

**FIGURE 5:**
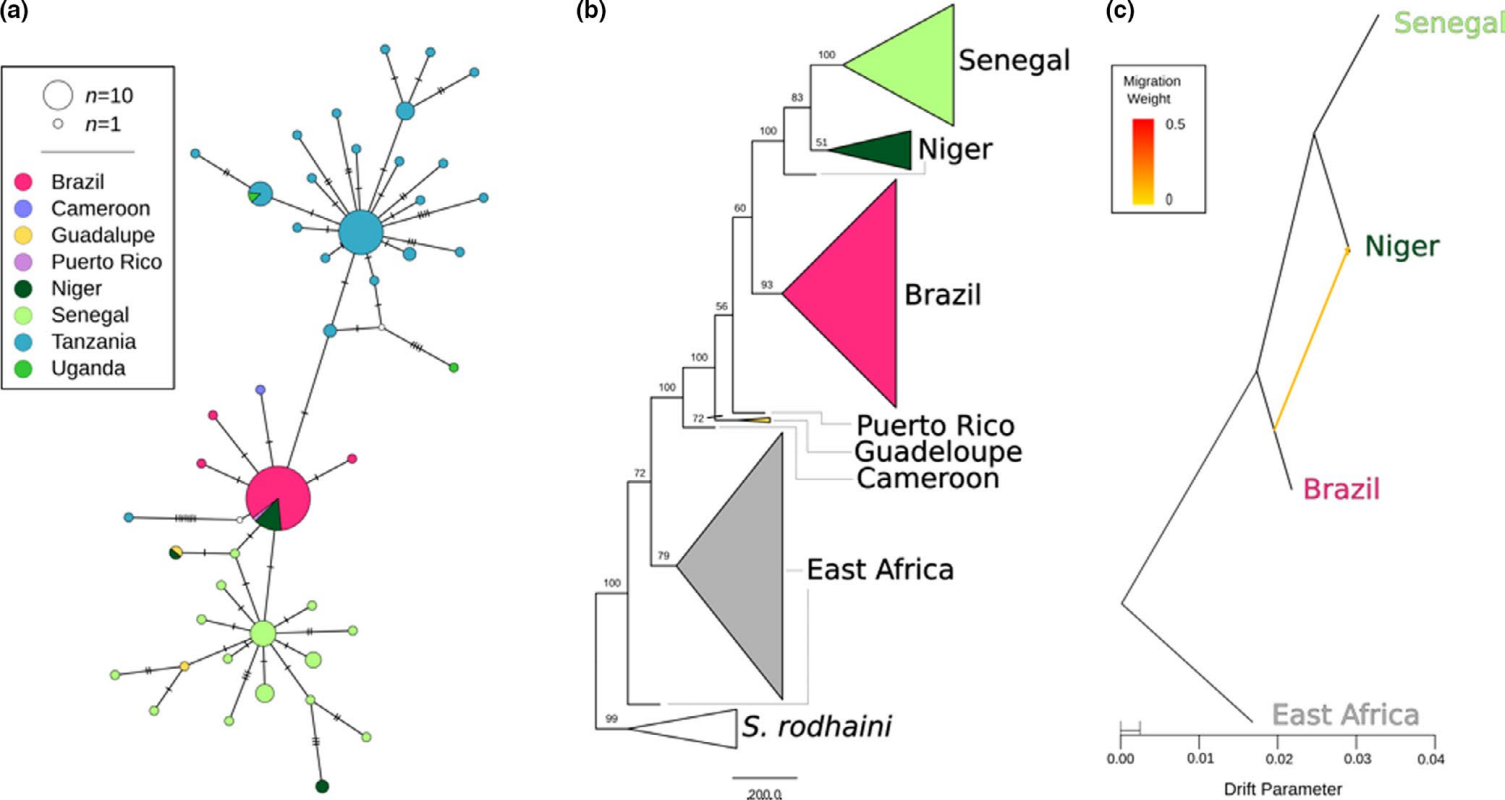
Phylogenetic relationships between *Schistosoma mansoni* populations. Multiple phylogenetic analyses and marker types were used to discern relationships between *S. mansoni* populations. (a) A median‐joining haplotype network was constructed from 815 variants across the mitochondria of all *S. mansoni* samples. (b) A coalescent‐based species tree from 100,819 parsimony‐informative single nucleotide variants with bootstrap values shown on each clade. Monophyletic populations are shown as a collapsed clade except in the case of East Africa which contains samples from Tanzania and Uganda. (c) A maximum‐likelihood phylogenetic network of autosomal variants identified a single, weak migration edge orientated from Brazil to Niger. All three analyses identify a relationship between Senegal, Niger and Brazil that excludes East African samples. The mitochondrial (a) and autosomal (c) networks both allow for direct relationship or allele sharing between Brazil and Niger. The species tree (b) indicates a strong relationship between Senegal and Niger that excludes Brazil (bootstrap percentage = 100)

We used three different phylogenetic methods to investigate the evolutionary relationships between populations (Figure 5). First, we generated a median‐joining network from 815 mitochondrial SNVs of which 477 where phylogenetically informative (Figure 5a). The haplotype network identified three major haplotypes roughly corresponding to the geographical partitioning of the samples. The haplogroups include an East African clade (Tanzania, Uganda), a Senegal group, and an intermediate haplogroup with samples from the Brazilian and Nigerien populations. Caribbean samples were not assigned to a single haplogroup. The single sample from Puerto Rico was associated with the major Brazilian and Nigerien haplotype, and the two Guadalupe samples tended to be more strongly associated with Senegalese haplotypes. Additionally, the sample from Cameroon was only a single step removed from the most common Brazilian haplotype.

A coalescent‐based species tree from 100,819 parsimony‐informative SNVs was generated with svd‐quartets (Figure 5b). Quartet sampling was limited to 100,00 quartets which sampled 0.43% of all distinct quartets present in the alignment. The final species tree was consistent with 84.7% of all the quartets sampled. Unlike the mitochondrial tree, samples fall into well‐supported clades corresponding to geography with two exceptions. Samples from East Africa and Niger formed independent paraphyletic clades. In both cases paraphyly was induced by a single individual. Bootstrap support was generally higher in the quartet species tree than in the mitochondrial tree. West African samples formed a well‐supported monophyletic clade with the Brazilian and Caribbean samples, indicating a shared origin for these parasites. Brazilian and Caribbean samples appear to have a polyphyletic origin within a larger clade containing West African parasites.

Finally, migration between populations was quantified with treemix (Figure 5c). We only examined populations with more than five individuals, which excluded Cameroonian and Caribbean samples from the analysis. The topologies linking the remaining populations (East Africa, Niger, Senegal and Brazil) in the treemix and species tree were identical. The species tree was slightly improved with the addition of a single migration edge from Brazil to Niger. This migration edge significantly improved the likelihood score of the topologies from 73.6026 to 73.7165 with an edge weight of 0.1 (*p* = .0081).

### 3.6 Selection

We used msprime to generate a set of neutrally evolving SNVs based on parameters specific to each of the sampled populations. These neutrally evolving SNVs were distributed across an 88.9‐Mb chromosome that was equal in size to *S. mansoni* chromosome 1 (HE601624.2). We then transposed the HE601624.2 exome annotation onto the simulated chromosome to extract “exome” data. This process was repeated 342 times per population to produce a set of neutrally evolving loci to use as controls when examining selection on actual samples. We used these neutral simulations to generate maximum (100%) threshold values for h‐scan and sweepfinder2 test statistics expected under neutrality (see below). The mean (*x̅*) simulated SNV count across all replicates was: *x̅*_Brazil_ =11,198 (range =10,874–11,552), *x̅*_Niger_ =8448 (range =8141–8706), *x̅*_Senegal_ =11,977 (range =11,642–12,309) and *x̅*_Tanzania_ =33,168 (range =32,575–33,741). The number of observed SNVs was between 72.2% and 89.7% of the mean number of simulated SNVs (Brazil =8947; Niger =6107; Senegal =9381; Tanzania =29,765). The reduced number of SNVs in the neutral data is probably due to the absence of selection that is presumed to be acting on the exome data sets.

**FIGURE 6:**
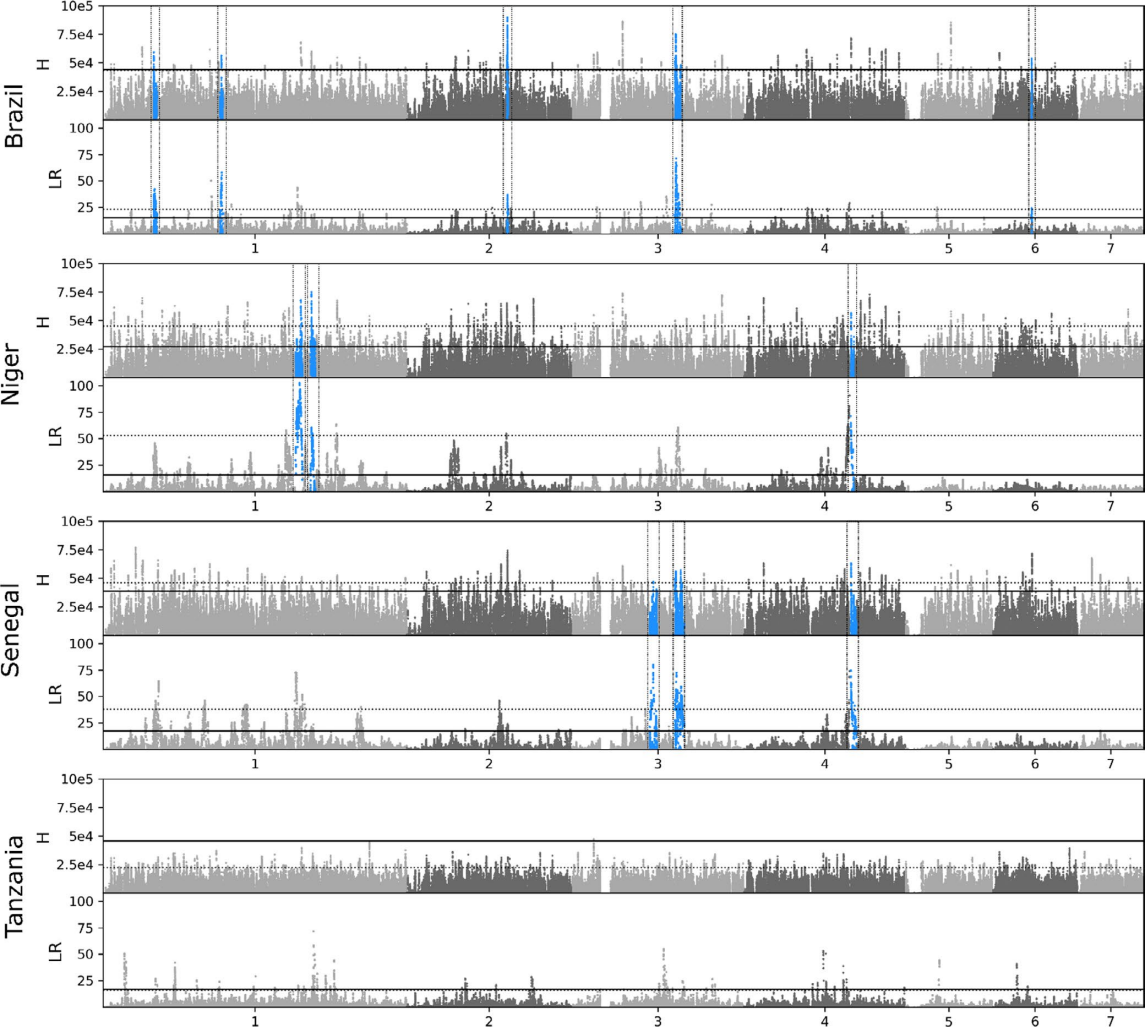
Positive selection across the *Schistosoma mansoni* genome. Selection across the *S. mansoni* genome was calculated with haplotype (*H*)‐ and allele frequency (likelihood ration; LR)‐based methods. Dotted lines represent positions with an *H* or LR value in the 99th percentile. The solid black line represents the maximum *H* or LR calculated from simulated data under neutral conditions. Regions of interest (blue, boxed) were identified by finding sites where the *H* and LR values were in the >99th percentile and were both greater than the maximum *H* or LR from simulated data. Once these sites were identified we combined variants within 333,333‐bp windows that showed signs of selection: an *H* or LR greater than the simulated threshold

The h‐scan and sweepfinder2 results are shown in Figure 6. pcadapt results are show in Figure S2. Each program uses a different methodology to detect sites under selection. h‐scan calculates *H* with homozygous tract length and the number of haplotypes to identify genome regions that have undergone selective sweeps from unphased variants. *H* values were highly variable for each population, even within windows smaller than 100 kb. In Tanzania only two of the 475,081 SNVs had *H* values higher than those generated from neutral simulations. sweepfinder2 calculates deviations from a neutral site frequency spectrum correcting for the possibility of background selection. An LR describes the probability of positive selection vs. neutral evolution and background selection within a designated window. sweepfinder2 was able to clearly define multiple peaks for each population when compared to h‐scan. Each of the four populations had regions greater than neutral expectations. pcadapt identifies SNVs significantly associated with population differentiation. In this analysis we found 442 SNV outliers, after multiple test correction, that were distributed across all seven autosomes at 127 loci. These regions were distributed across 280.2 Mb. *F*_ST_ of the pcadapt outliers (*F*_ST_ =0.544) was significantly higher than in the remaining population outliers (*F*_ST_ =0.195).

We defined “putative regions of selection” as those that have most likely experienced positive selection. These regions contain variants (i) with both *H* and LR values in the 99th percentile, (ii) are greater than the neutral thresholds and (iii) have a signal of population‐specific directional selection. All SNVs meeting one or more of these criteria are listed in Table S4.

Our results recovered five, three and three putative selected regions in Brazil, Niger and Senegal respectively (Figure 6; Table S5). Information regarding the number of regions, SNVs and genes identified are presented in Tables 4 and 5. *π* (Figure S3) and Tajima's *D* (Figure S4) were depressed in these regions compared to genome‐wide values (Table S3), which is consistent with loci experiencing selection. On average the size of each region was relatively small (1,395,643 bp) and in two instances these sites were shared between populations. The Brazilian and Senegalese populations shared a site on chromosome 3 at HE601626.2:30,092,830:31,936,551, while the Nigerien and Senegalese populations shared a peak on chromosome 4 at HE601627.2:31,216,154:32,138,352. We did not recover any regions or sites of interest in the Tanzanian population in large part because only two of 475,081 sites had higher *H* values the largest *H* from neutral simulations (*H* = 45,532.9). These variants are on chromosome 3 at HE601626.2:6,500,422 and HE601626.2:6,503,809 and are adjacent to each other in our filtered SNV data set.

**TABLE 4:** Number of SNPs and regions identified in genome‐wide scans for selection

	Brazil	Niger	Senegal	Tanzania
Outlier SNVs (h‐scan)	4366	44,024	9182	2
Outlier SNVs (sweepfinder)	12,871	41,525	23,379	4288
Merged regions (1/3 Mb)	113	250	168	46
99th Percentile SNPs	703	239	176	0
Outlier SNPs (pcadapt)^a^	442	442	442	Na
Putative regions under selection	5	3	3	0
Genes in selected regions	116	112	157	0
Genes w/99th percentile SNPs	10	5	7	0

Abbreviations: SNP, single nucleotide polymorphism; SNV, single nucleotide variant.

^a^ Outlier SNPs are those that are greater than the maximum value derived from neutrally simulated data.

**TABLE 5:** Genes in *Schistosoma mansoni* populations with the strongest signals of directional selection

Gene ID	UniProtKB accession	UniProtKB description	HHsearch annotation
Brazil			
Smp_060090	G4VAJ9	40S ribosomal protein S12	Ribosomal protein S12e ribosome
Smp_073680	G4V701	Putative TATA‐box binding protein	DNA‐directed RNA polymerase II subunit
Smp_123510	A0A5K4EKW3	Vacuolar protein sorting‐associated protein 16 homolog	Vps16_C
Smp_123520^a^	A0A3Q0KKK4	Putative RNA M^5^U methyltransferase	rRNA (Uracil‐5‐)‐methyltransferase RumA
Smp_123570	A0A3Q0KKM5	BHLH domain‐containing protein	Aryl hydrocarbon receptor nuclear translocator
Smp_123590	A0A5K4EL22	Uncharacterized protein	Swi5‐dependent recombination DNA repair protein
Smp_148460	A0A3Q0KPP3	Putative neurofibromin	GAP‐related domain of neurofibromin
Smp_162000	A0A5K4ETP0	UBR‐type domain‐containing protein	E3_UbLigase_R4
Smp_246630	A0A5K4F427	UBC core domain‐containing protein	E2 Ubiquitin conjugating enzyme
Smp_341570	A0A5K4FC62	Uncharacterized protein	Uncharacterized protein family UPF0183
Niger			
Smp_008230	G4V7H8	Putative rab‐18	di‐Ras2
Smp_126620	A0A3Q0KL42	Uncharacterized protein	Ligand‐binding domain of low‐density lipoprotein receptor
Smp_165060	A0A3Q0KRY3	Uncharacterized protein	Uncharacterized protein
Smp_167890^b^	Q6BC90	Peptide‐methionine (*R*)‐S‐oxide reductase	C‐terminal MsrB domain of methionine sulphoxide reductase PilB
Smp_313490^b^	A0A5K4F3H5	Uncharacterized protein	Transforming protein RhoA, Rho‐associated, coiled‐coil
Senegal			
Smp_070780	G4VEM1	UDP‐glucose 4‐epimerase	Uridine diphosphogalactose‐4‐epimerase
Smp_123440	A0A3Q0KKL2	Putative fad oxidoreductase	d‐amino‐acid oxidase
Smp_123520^a^	A0A3Q0KKK4	Putative RNA M^5^U methyltransferase	rRNA (Uracil‐5‐)‐methyltransferase RumA
Smp_164560	A0A3Q0KRR1	Uncharacterized protein	Na
Smp_167890^b^	Q6BC90	Peptide‐methionine (R)‐S‐oxide reductase	C‐terminal MsrB domain of methionine sulfoxide reductase PilB
Smp_213150	A0A5K4EZI5	Uncharacterized protein	Ribonucleases P/MRP protein subunit POP1
Smp_313490^b^	A0A5K4F3H5	Uncharacterized protein	Transforming protein RhoA, Rho‐associated, coiled‐coil

Note

UniProtKB descriptions are from release 2020_06. HHsearch annotations are from Le Clec'h et al. (2021).

^a^ Shared between Brazil and Senegal.

^b^ Shared between Niger and Senegal.

We identified 116–157 genes within “putative selected regions” in the Brazilian, Nigerien and Senegalese populations (Table S6). Within these populations, 10, five and seven genes contain SNVs meeting the 99th percentile. Several genes identified in these regions were shared between populations. Brazil and Senegal shared 48 genes in target regions, and Senegal and Niger shared 22 genes in target regions. Three genes with 99th percentile SNVs were shared between populations: Smp_313490 (Uncharacterized protein) and Smp_167890 (Peptide‐methionine (*R*)‐S‐oxide reductase; Niger and Senegal), Smp_123520 (Putative RNA M^5^U methyltransferase; Brazil and Senegal).

## 4 DISCUSSION

We examined the impact of human‐mediated dispersal of *Schistosoma mansoni* during the Trans‐Atlantic slave trade. Previous analyses with *S. mansoni* used mitochondrial data or had limited sampling from the Americas (Crellen et al., 2016; Morgan et al., 2005; Webster et al., 2013). To build upon these previous studies, we included 135 exome sequences available from natural populations in Brazil (*n* = 45), Niger (*n* = 10), Senegal (*n* = 25) and Tanzania (*n* = 55) which we combined with the existing genome sequences from Cameroon (*n* = 1), the Caribbean (*n* = 4), Senegal (*n* = 1) and Uganda (*n* = 2) (Berriman et al., 2009; Chevalier et al., 2016; Crellen et al., 2016). We used these data to explore the *S. mansoni* expansion across Africa, examine parasite colonization of the Americas, quantify signatures of selection during colonization and detect hybridization with *Schistosoma rodhaini*, a closely related parasite utilizing a rodent host.

### 4.1 Elevated East African diversity and *S. mansoni* expansion across Africa

A striking result from this study is the dramatic reduction in genetic diversity between East and West Africa. Sequence summary statistics indicate that the East African population has two‐ to three‐fold greater nucleotide diversity (*π*), larger *N*_e_ and greater mitochondrial diversity than the other populations (Table 1; Figure 5a). Phylogenetic analyses rooted with *S. rodhaini* clearly indicate that the East African *S. mansoni* samples from Tanzania and Uganda are sister to a clade containing all other *S. mansoni*, including those from the Caribbean, Brazil, Senegal, Niger and Cameroon. Our data support previous work from whole genomes, mitochondrial genes and microsatellites suggesting that *S. mansoni* emerged in East Africa (Crellen et al., 2016; Morgan et al., 2005; Webster et al., 2013). In addition, our estimates of *N*_e_ are within a range book‐ended by other whole genome studies (Berger et al., 2021; Crellen et al., 2016).

It is possible that drug treatment differences between localities have an unseen impact on these results. Mass drug administration (MDA) has been shown to reduce, and in some cases even eliminate, transmission of schistosomes. However, it is not uncommon for schistosomes to persist in populations even in the midst of significant MDA efforts. In these areas, MDA has minimal impacts on the genetic diversity of schistosome populations (Berger et al., 2021; Faust et al., 2019; Gower et al., 2017; Huyse et al., 2013; Lelo et al., 2014).

The rapid decay in LD observed in East Africa compared with West African and American populations provides further evidence that East African *S. mansoni* populations are ancestral. Similar reductions in the rate of LD decay have been observed in humans and malaria parasites, outside of their ancestral Africa range (Anderson et al., 2000; Gurdasani et al., 2015; Neafsey et al., 2008). Rapid breakdown in LD also has important practical applications for genome‐wide association analyses (GWAS), because it allows mapping of phenotypic traits to very narrow regions of the genome (Mackay & Huang, 2018). There are multiple biomedically important traits of interest that vary in *S. mansoni* populations, including drug susceptibility or resistance, host specificity and cercarial production (Anderson et al., 2018). We recently used GWAS for mapping resistance to the first‐line drug (Praziquantel) in laboratory schistosome populations (Le Clec’h et al., 2021). Most laboratory *S. mansoni* populations tested were from South America, where LD decays relatively slowly: these are not ideal for GWAS. Establishment of laboratory *S. mansoni* populations from East Africa, or GWAS analyses using parasites directly from the field would be valuable for future GWAS with *S. mansoni*.

There is minimal allele sharing or migration between East African and other *S. mansoni* populations. *F*_ST_ comparisons that include Tanzania are greater than other comparisons (with Tanzania *F*_ST_ = 0.355; excluding Tanzania *F*_ST_ = 0.206). East African populations are among the most strongly differentiated populations in the PCAs (Figure 3b) and the East African population component in admixture analyses is absent, or at minimal levels, in other *S. mansoni* populations. Finally, mitochondrial haplotypes in Uganda and Tanzania form a distinct haplogroup from other *S. mansoni* populations. These data indicate that while East Africa is the probable origin of *S. mansoni*, migration or allele sharing between East Africa and other populations is restricted.

We do not expect that human movement is a major barrier between East and West African schistosome populations. However, differences in snail–schistosome compatibility in East and West Africa may provide barriers to gene flow. This is seen in multiple host–parasite systems, including *Daphnia*–microsporidia (Ebert, 1994), and trematode infections of snails (Lively, 1989) and minnows (Ballabeni & Ward, 1993). Strong host‐specificity exists within the *Biomphalaria* and *S. mansoni* system (Mitta et al., 2017; Theron et al., 2014; Webster & Woolhouse, 1998) and a review shows that compatibility is greater between sympatric *Biomphalaria*–*S. mansoni* combinations (Morand et al., 1996). Sympatric schistosome–snail combinations result in rapid immune suppression and rapid parasite development, while allopatric schistosome–snail combinations result in a slower immune cell proliferation and a nonspecific generalized immune response which reduced parasite growth and establishment (Portet et al., 2019). There is a developing understanding of *Biomphalaria* phylogenetics (Jorgenson et al., 2007), phylogeography (Dejong et al., 2003) and compatibility relationships among East African snail species (*B sudanica*, *B. pffeiferi* and *B. choanomphala*) and *S. mansoni* (Mutuku et al., 2017, 2021), but further research is needed to understand the compatibility of allopatric snail–schistosome combinations from East and West Africa. We suggest that the presence of fine‐scale geographical structure of *Biomphalaria* populations (Webster et al., 2001) and local adaptation in sympatric *Biomphalaria*–schistosome combinations may limit parasite geneflow between East and West Africa.

PCAs differentiate *S. mansoni* populations on an East‐to‐West gradient along PC2 (Figure 3b). Mitochondrial haplotypes in Senegal and Cameroon are intermediate to those in Tanzania and Senegal (Figure 5a). The species tree from autosomal SNV data (Figure 5a) indicates that *S. mansoni* is in a series of nested, well‐supported clades from East Africa (Tanzania+Uganda), to Cameroon and to West Africa (Niger+Senegal). *F*_ST_ and *r* (Mantel) values between Tanzania, Niger and Senegal reflect increasing isolation with distance across Africa. These observations combined with the origination of *S. mansoni* in East Africa confirms an East‐to‐West, stepwise expansion of *S. mansoni* from Tanzania and Uganda → Cameroon → Niger → Senegal (Crellen et al., 2016; Morgan et al., 2005; Webster et al., 2013).

### 4.2 Does hybridization between *S. rodhaini* and *S. mansoni* contribute to elevated East African diversity

Several closely related *Schistosoma* species are able to hybridize and produce viable offspring, as confirmed via experimental rodent infections. The potential for hybridization between animal and human *Schistosoma* species is a significant public health concern (Borlase et al., 2021; Leger & Webster, 2017; Léger et al., 2020; Stothard et al., 2020). Our group, and others, have recently shown that ancient hybridization and adaptive introgression has resulted in the transfer of genes from the livestock species *Schistosoma bovis* into *S. haematobium*: West African *S. haematobium* genomes contain 3%–8% introgressed *S. bovis* sequences and *S. bovis* alleles have reached a high frequency in some genome regions (Platt et al., 2019; Rey, Toulza, et al., 2021). The sister species of *S. mansoni*, *S. rodhaini*, parasitizes rodents and is primarily located in eastern Africa (Rey, Webster, et al., 2021). *S. mansoni* and *S. rodhaini* have been shown to readily hybridize in and produce fertile offspring in the laboratory (Théron, 1989). Natural hybrids have been reported in Kenya and Tanzania (Morgan et al., 2003; Steinauer, Hanelt, et al., 2008), although hybrids have only been detected from their snail intermediate host and never encountered in the mammalian hosts, humans and rodents (Rey, Webster, et al., 2021). We were unable to find evidence of hybridization in 55 samples collected from Tanzania. Both species are clearly separated in genotypic space with differences between the species accounting for the largest component in the PCA (Figure 3a; PC1 = 34.7% variation), and *F*_ST_ between *S. rodhaini* and *S. mansoni* populations is very high (*F*_ST_ = 0.912). admixture analyses also clearly differentiated *S. rodhaini* from all other *S. mansoni* populations (Figure 4) and we were unable to identify admixture signal between *S. rodhaini* and the Tanzanian population with genome‐wide statistics including *D*, *D*_3_ and *F*_3_ (Table 3).

Hybridization between these two species is thought to be rare (≤7.2%) (Morgan et al., 2003; Rey, Webster, et al., 2021; Steinauer, Hanelt, et al., 2008; Steinauer, Mwangi, et al., 2008). Our sample size may not be large enough to identify rare hybrids. Furthermore, we analysed exome (coding) data, which may underrepresent introgressed alleles if they are selected against. Finally, the *S. rodhaini* samples are primarily from a single laboratory population that may not be representative of natural populations. These caveats aside, our analyses clearly failed to identify recent hybridization between *S. mansoni* and *S. rodhaini*. We conclude that *S. rodhaini* introgression does not contribute to the high genetic diversity in our Tanzanian *S. mansoni* samples.

### 4.3 Expansion into the Americas

Previous work has shown that *S. mansoni* was exported from Africa to the Americas during the Trans‐Atlantic slave trade (Crellen et al., 2016; Desprès et al., 1993; Files, 1951; Fletcher et al., 1981; Morgan et al., 2005; Webster et al., 2013). Here, we use genomic data to investigate the probable source population(s), number of introductions, evidence for bottlenecks and parasite adaptation during colonization.

#### 4.3.1 Source populations

Of the two West African populations sampled (Niger and Senegal), our results support stronger relationships between Brazil and Niger, than with Senegal. While the species tree (Figure 5b) appears to rule out Niger or Senegal as the direct source population for Brazilian *S. mansoni*, there is evidence of allele sharing between the Nigerian and Brazil populations. First, the dominant mitochondrial DNA (mtDNA) haplotype in Brazilian and Nigerien samples is shared (Figure 5a). Second, every Nigerien population contains at least 10.1% of the Brazilian component, as shown in the admixture analyses (mean 15.8%; Figure 4). Third, Niger and Brazil are more closely associated with each other along a genotypic continuum, represented by PC2, than Brazil is to other African populations (Figure 3b). Finally, treemix identified a single weak migration edge between Brazil and Niger (Figure 5c), confirming a relationship between these two populations.

**FIGURE 7:**
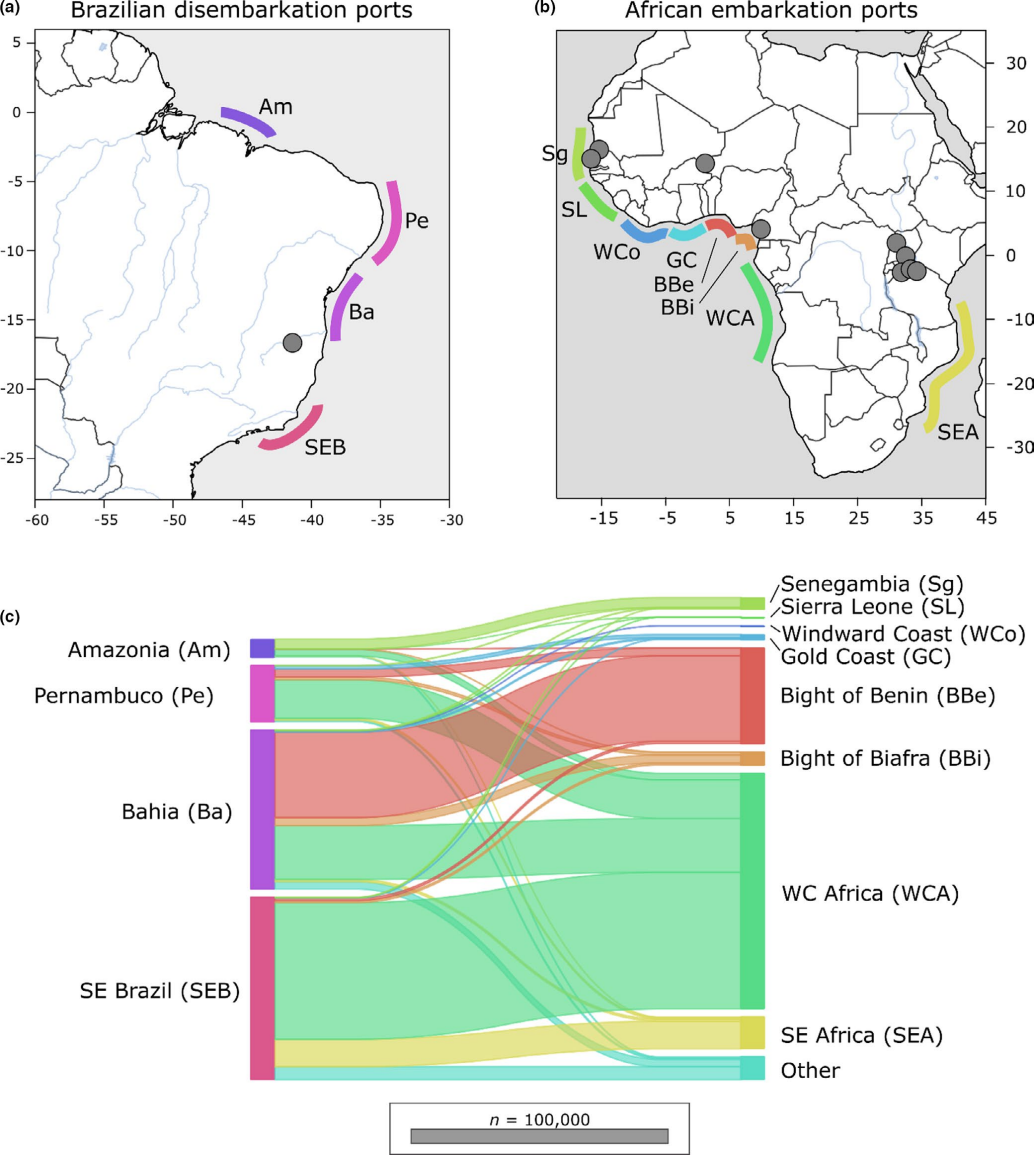
Export and importation of slaves between Africa and Brazil. During the Trans‐Atlantic slave trade, 12 million people were trafficked from Africa. More than 3.5 million were exported into Brazil at major ports along the east coast. Most slaves originated from regions along the West African coast from Benin to Angola. General locations of major embarkation and disembarkation ports are shown along the (a) Brazilian and (b) Africa coastlines. (c) The movement of people between Brazilian and African ports is shows with horizontal lines. Line width is proportional to the number of migrants. The Brazilian samples in this study were collected from Ponto dos Volantes in Minas Gerais, Brazil, between the Ports of Bahaia and South East Brazil. The primary embarkation points for Bahaia and South East Brazil were in West Central Africa and the Bight of Benin. These regions are a probable source for the *S. mansoni* collected in Ponto dos Volantes. Collection localities for *S. mansoni* are represented by grey dots. Slave exportation data are from the Slave Voyages Database (2009)

A simple hypothesis from the data is that, assuming a general east‐to‐west expansion holds at finer geographical scales, the source population that was eventually exported to Brazil is probably located somewhere between Benin and Angola. These countries fell within the Bight of Benin, Bight of Biafra and West Central Africa slave trading regions (Figure 7). Our Brazilian samples were collected in Ponto dos Volantes in Minas Gerais, Brazil. This location is relatively equidistant from major slave ports in Bahia (527 km) and around Rio de Janeiro (706 km). In all, more than 3.5 million slaves were transported to Brazil (Table S7; Slave Voyages Database, 2009). Of all slaves taken to Brazil, 82% disembarked at ports in either Bahia (1.3 million) or Rio de Janerio (1.5 million). In Africa, slave‐exporting markets in the Bights of Benin and Biafra and West Central Africa were responsible for 53.6%, 5.0% and 33.8% of people exported to Bahia and 1.3%, 1.1% and 74.9% of those exported to southeast Brazilian ports (Slave Voyages Database, 2009). Taken together these data imply that the Brazilian population we sampled in Ponto dos Volantes probably originated from markets in the Bight of Benin or West Central Africa. In our phylogenetic analyses, the Brazilian population falls within a clade containing samples from Senegal, Niger and the Caribbean but excludes the single Cameroonian sample. If the Cameroonian sample is representative of the Cameroonian population, then it may be that Cameroon and Niger represent the eastern and western limits of the unrepresented source population. This area more closely aligns with the Bight of Benin, a region containing parts of Nigeria and Benin. Additional samples from these regions are needed to test this hypothesis. It is also important to note that the Brazilian samples here represent a single geographical location (Ponto dos Volantes) and that the source for this population may not extrapolate to larger regions, or even to other locations in Brazil.

#### 4.3.2 No evidence for population bottlenecks during colonization

Previous mtDNA analyses have shown reduced diversity in South American parasites (Desprès et al., 1993; Fletcher et al., 1981; Morgan et al., 2005; Webster et al., 2013), suggesting bottlenecks during colonization. Our data are consistent with this, showing a two‐ to three‐fold reduction in mtDNA in Brazil compared to West African parasites. However, genome‐wide summary statistics of autosomal sequence data tell a different story, and provide no evidence for population bottlenecks associated with *S. mansoni* introduction and establishment in Ponto dos Volantes, Brazil (Table 1). Nucleotide diversity, measured by *π*, was higher in Brazil than either of the West African populations (Niger or Senegal), perhaps because the Brazilian population is derived from multiple West African source populations. The Brazilian *N*_e_ was roughly the same as for Niger and comparable to Senegal. Mean Tajima's *D* calculated from genome‐wide SNV data is negative in each of the African populations and highest in the Caribbean samples (mean Tajima's *D* = 0.929; Table S2). Tajima's *D* values in our study using exome SNV data from different regions of Niger, Senegal and Tanzania were comparable with values calculated using whole genome SNV data from Ugandan *S. mansoni* (Berger et al., 2021). By contrast, mean Tajima's *D* in Brazilian samples is close to 0 (mean Tajima's *D* = 0.034; 95% CI [−0.18 to −0.085]) and is significantly greater than mean Tajima's *D* in the African samples (*t* test *p* < .001; Table S2).

The nuclear genomic data clearly suggest that the establishment of *S. mansoni* in Brazil was not associated with a significant population bottleneck and had minimal impacts on genome‐wide levels of genetic diversity. The discrepancy between mtDNA and nuclear DNA may stem from two sources. First, mtDNA has an effective population size one‐quarter that of nuclear genes (Birky et al., 1983), and can potentially provide a more sensitive indicator of bottlenecks. Second, and perhaps more critical, mtDNA constitutes a single marker, so may poorly reflect population history (Anderson, 2001). Extensive laboratory passage may also result in bias in population summary statistics. For example, the Caribbean samples examined have undergone 2–15 generations of laboratory passage, which is probably responsible for the elevated Tajima's *D* in this population (Crellen et al., 2016).

#### 4.3.3 Number of introductions

All Brazilian and Caribbean samples are paraphyletic and fall between the Cameroonian sample and West African clade in Figure 5b. The relationships among these samples are resolved but not supported outside of a monophyletic clade containing the Brazilian samples. As a result, the autosomal phylogeny by itself does not conclusively support one or multiple introductions into the Americas. The two Caribbean samples from Guadeloupe island contain unique mitochondrial haplotypes absent from Brazil (Figure 5a); however, this is, at best, only weak evidence for independent introductions into Brazil and the Caribbean from the data we have available. The higher autosomal diversity of Brazilian *S. mansoni* compared with two sampled West African populations provides additional indirect evidence for multiple origins.

*Plasmodium falciparum* and *W. bancrofti* became established in the Americas at the same time as *S. mansoni*, and without apparent bottlenecks (Small et al., 2019; Yalcindag et al., 2012). In both cases it is hypothesized that high levels of diversity were maintained by recurring introductions from multiple sources (Rodrigues et al., 2018; Small et al., 2019; Yalcindag et al., 2012). More than 3.5 million slaves were transported to Brazil from across the continent of Africa (Table S7; Slave Voyages Database, 2009). *S. mansoni* prevalence varies widely between sites in western and central Africa, with estimates ranging from 0.3% (Gambia; Sanneh et al., 2017) to 89% (Democratic Republic of the Congo; Kabongo et al., 2018). Assuming *S. mansoni* prevalence during the period of the Atlantic Slave Trade was comparable to current levels and that infected people contained multiple *S. mansoni* individuals (genotypes; Van den Broeck et al., 2014), it is plausible that hundreds of thousands, and more probably millions, of reproductively viable *S. mansoni* were introduced into Brazil from across Africa. As a result, individual genotypes that may have been separated by thousands of miles in Africa were brought into close contact in the Americas and would lead to higher levels of diversity there than in any single population in Africa. The lack of bottlenecks during establishment of *S. mansoni* in the New World is consistent with compatibility between West African *S. mansoni* and South American *Biompahalaria* species. We note that this scenario can be directly tested. It is possible to experimentally explore this biological invasion using experimental infection of laboratory‐maintained South American snail populations with miracidia derived from West Africa.

#### 4.3.4 Adaptation during colonization

We hypothesized that *S. mansoni* introduced into the Americas would have been exposed to novel selective pressures as they adapted to new biotic and abiotic challenges. For example, the *S. mansoni* life cycle requires an intermediate snail host in which miracidia mature into cercariae that are capable of infecting humans. Previous work has shown the snail immune response is greater when exposed to sympatric parasite strains (Portet et al., 2019) and, in general, sympatric host/parasite combinations to be more compatible (Morand et al., 1996). In Africa, *S. mansoni* use *Biomphalaria alexandrina*, *B. camerunensis*, *B. choanomphala*, *B. pfeifferi*, *B. stanleyi* and/or *B. sudanica* as the snail host, none of which are present in the Americas (Figure 1). Instead, these parasites have adapted to using different *Biomphalaria* hosts, including *B. glabrata*, *B. straminea* and *B. tenagophila* (Hailegebriel et al., 2020; Kengne‐Fokam et al., 2018; Vidigal et al., 2000). We examined exomic SNV data to identify genes and larger regions of the genome under selection at a finer scale and identified zero to five putative regions of selection from each of the major populations (Table 4).

In the Brazilian samples, we identified five putative selected regions that contain 126 genes (Table S6). *π* and Tajima's *D* were significantly reduced in these five regions compared to genome‐wide averages, which is expected if these loci are, or have been, under selection (Table S3). One region is shared between the Brazilian and Senegalese populations. Forty‐six genes fall within this Senegal–Brazil overlapping region, leaving 80 genes and four loci that are probably experiencing population‐specific positive selection. Even within the group of 80 genes, there are nine with strong signals of selection (Table 5). These genes contained variants with *H* and LR values in the 99th percentile in addition to being greater than the threshold defined by neutral simulations. Several genes within this group are associated with housekeeping functions, including transcription and protein degradation (Smp_060090, 40S ribosomal protein S12; Smp_162000, UBR‐type domain‐containing protein; Smp_246630, UBC core domain‐containing protein). Two uncharacterized proteins were identified (Smp_341570 andSmp_123590) but we are not able to speculate on their function. Of particular interest are two possible transcription factors, an uncharacterized protein containing a helix, loop, helix domain (Smp_123570, BHLH domain‐containing protein) and a putative TATA‐box binding protein (Smp_073680, Putative TATA‐box binding protein). It is possible that adaptation to the Brazilian environment was driven by changes in gene expression, but more work is needed to understand the potential role of these loci in adaptation to the Americas.

#### 4.3.5 Selection on African *S. mansoni*

We examined selection on African *S. mansoni* as part of the process to identify unique signals of selection in the Brazilian population. We identified 112 and 157 genes under selection in three regions each for the Nigerien and Senegalese populations (Table 4). One of the three regions, and 22 genes, was shared between Niger and Senegal. We failed to identify any regions of selection in Tanzania using our combined criteria, but results from individual tests of selection (h‐scan, sweepfinder2) did overlap at nine of 25 regions (Table S8) identified in a large Ugandan population (Berger et al., 2021). These regions were identified using a variety of within‐ (iHS) and between‐population (*F*_ST_, XP‐EHH) tests on miracidia isolated from two Ugandan locations with differing histories of praziquantel treatment.

## 5 CONCLUSIONS

Our analyses confirmed an east‐to‐west expansion of *Schistosoma mansoni* across Africa. Sometime during this expansion one or more Central African population(s), probably located between Angola and Benin, were transported to Brazil. Genome‐wide signatures of diversity, measures of allele frequencies (Tajima's *D*) and estimates of *N*_e_ are comparable between *S. mansoni* in Brazil, Niger and Senegal and do not imply the presence of a bottleneck during the establishment of *S. mansoni* in Brazil. We did find five genome regions under selection in Brazil, four of which are population‐specific. In total, 80 genes fall within these regions and may be associated with *S. mansoni's* adaptation to novel selection pressures associated with the Americas. We identified nine genes with the strongest signals of selection that are candidates for future experimental work.

## CONFLICT OF INTEREST

The authors declare no competing interests.

## AUTHOR CONTRIBUTIONS

Study design: F.D.C., T.J.A. and W.L.C; formal analysis: F.D.C., R.N.P. and T.J.A.; writing, original draft: R.N.P. and T.J.A.; reviewing and editing: A.G., A.E., B.W., D.R., F.D.C., G.O., J.P.W., M.M, P.T.L., R.R.dA., R.N.P., S.K., T.J.A. and W.L.C.; investigationF.D.C., M.M. and W.L.C.; resources were provided by F.D.C. and T.J.A.

### OPEN RESEARCH BADGES

This article has earned an Open Data Badge for making publicly available the digitally‐shareable data necessary to reproduce the reported results. The data is available at https://doi.org/10.5061/dryad.dv41ns209.

## Supporting information

Fig S1

Table S1

## Data Availability

Data used in this paper were previously published (Berriman et al., 2009; Chevalier et al., 2019; Crellen et al., 2016; International Helminth Genomes Consortium, 2019; Le Clec'h et al., 2021) under multiple NCBI BioProject (PRJNA439266, PRJNA560070, PRJEB522, PRJEB526, PRJNA743359 and PRJNA773498) and NCBI Short Read Archive (ERR046038, ERR103049, ERR103050, ERR119614, ERR119615, ERX284221, ERR310938, ERR539846, ERR539847, ERR539848, and ERR9974) accessions. Code availability: Scripts, notebooks, and environmental yaml files are available at https://github.com/nealplatt/sch_man_nwinvasion/releases/tag/v0.2 (last accessed October 21, 2021) or https://doi.org/10.5281/zenodo.5590460 (last accessed October 21, 2021).
