# Supplementary material for: Genomic analysis of a parasite invasion: Colonization of the Americas by the blood fluke Schistosoma mansoni
Source: Mol Ecol. 2022 Feb 25;31(8):2242–63. doi: 10.1111/mec.16395 (PMC9305930; doi:10.1111/mec.16395)
Supplement: Supplementary file 1 — Fig S1 [file MEC-31-2242-s002.docx]

**Supplemental Information for:**

**Genomic analysis of a parasite invasion: colonization of the Americas by the blood fluke, Schistosoma mansoni**

Roy N. Platt II*, Winka Le Clec'h*, Frédéric D. Chevalier*, Marina McDew-White, Philip T. LoVerde, Rafael R. de Assis, Guilherme Oliveira, Safari Kinunghi, Amadou Garba Djirmay, Michelle L. Steinauer, Anouk Gouvras, Muriel Rabone, Fiona Allan, Bonnie L. Webster, Joanne P. Webster, Aidan Emery, David Rollinson, Timothy J. C. Anderson

**Table of Contents:**

| **Supplemental Figure 1. Identifying regions of selection** | Page 2 |
| --- | --- |
| **Supplemental Figure 2. Directional selection across three *S. mansoni* populations** | Page 3 |
| **Supplemental Figure 3. Nucleotide diversity (π) across the genome** | Page 4 |
| **Supplemental Figure 4. Tajma’s D across the genome.** | Page 5 |

**
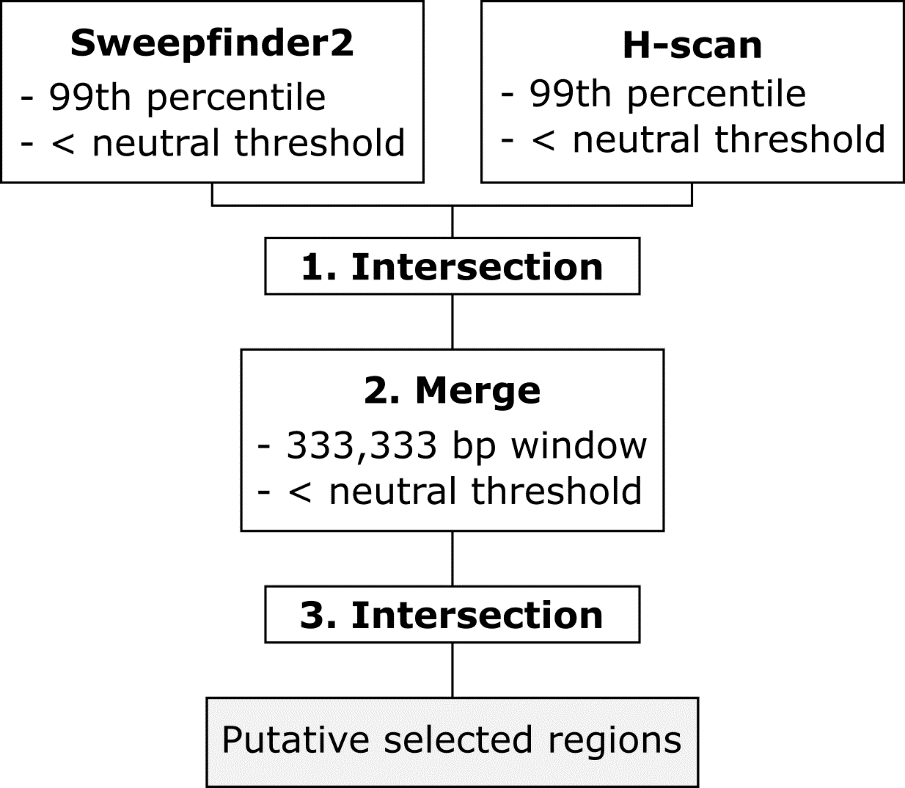
**

**Supplemental Figure 1. Identifying regions of selection.**  Flow chart shows how three separate analyses were combined to identify regions most likely experiencing positive selection. These regions are referred to as “putative selected regions”. We established neutral thresholds by simulating neutrally evolving SNVs for each population. The simulated data was then run through H-Scan and Sweepfinder2 to determine the maximum expected values from neutral data for each analysis.


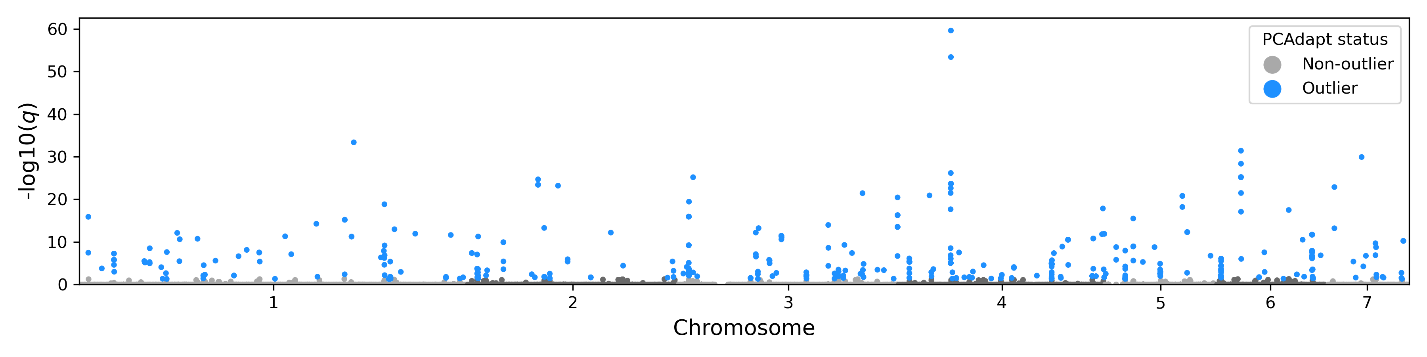
**Supplemental Figure 2. Directional selection across three *S. mansoni* populations.**  Population specific, directional selection was estimated at each single nucleotide variant (SNV) across the exome using pcadapt v4.3.3 (Luu et al., 2017). We examined three *S. mansoni* populations including indiviudals from Niger, Senegal, and Brazil. Outlier SNVs were identified after multiple test correction (Bonferroni) and α = 0.05.


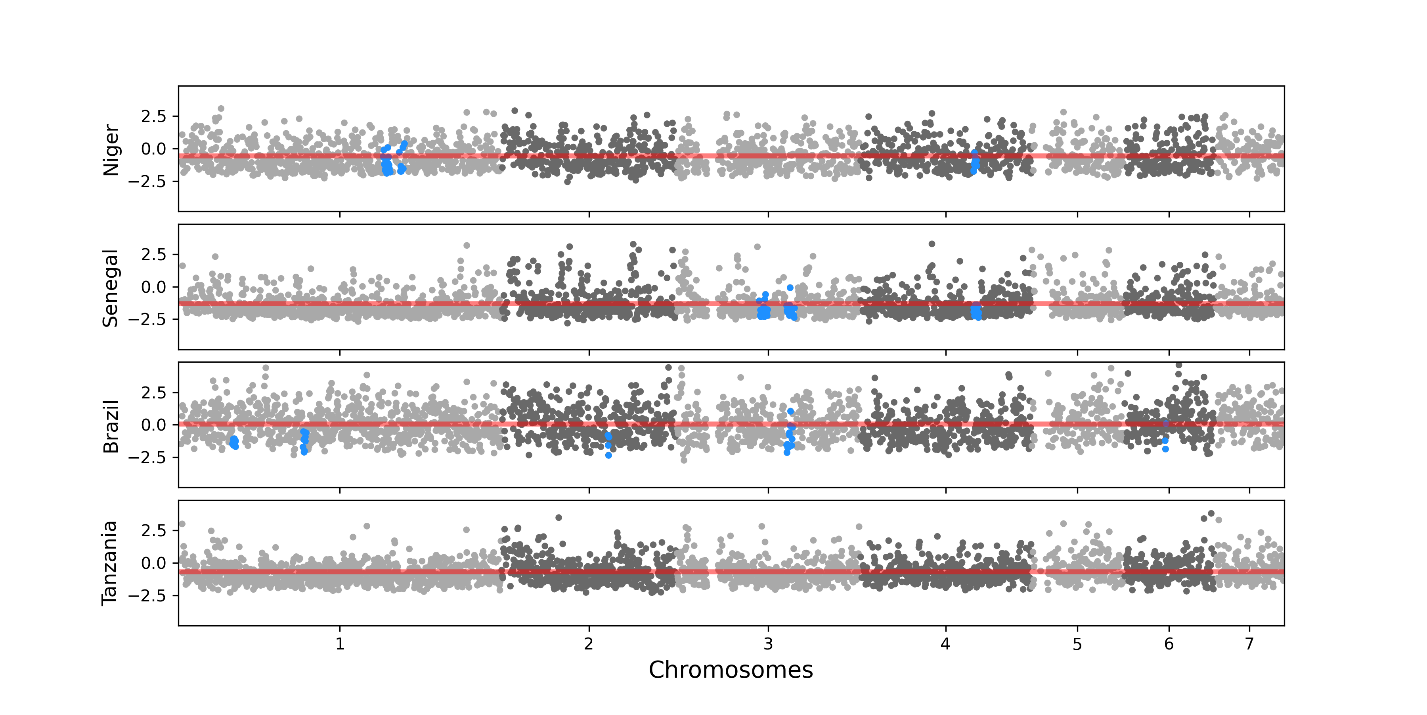
**Supplemental Figure 3. Nucleotide diversity (π) across the genome.** π was measured across the genome in each *S. mansoni* population. Average π for each population is indicated by the red line. π in regions identified as putative regions of selection are shown in blue. π was significantly lower in putative regions of selection than expected based on genome-wide averages in Niger, Senegal, and Brazil. We did not identify any putative regions of selection in the Tanzanian population.


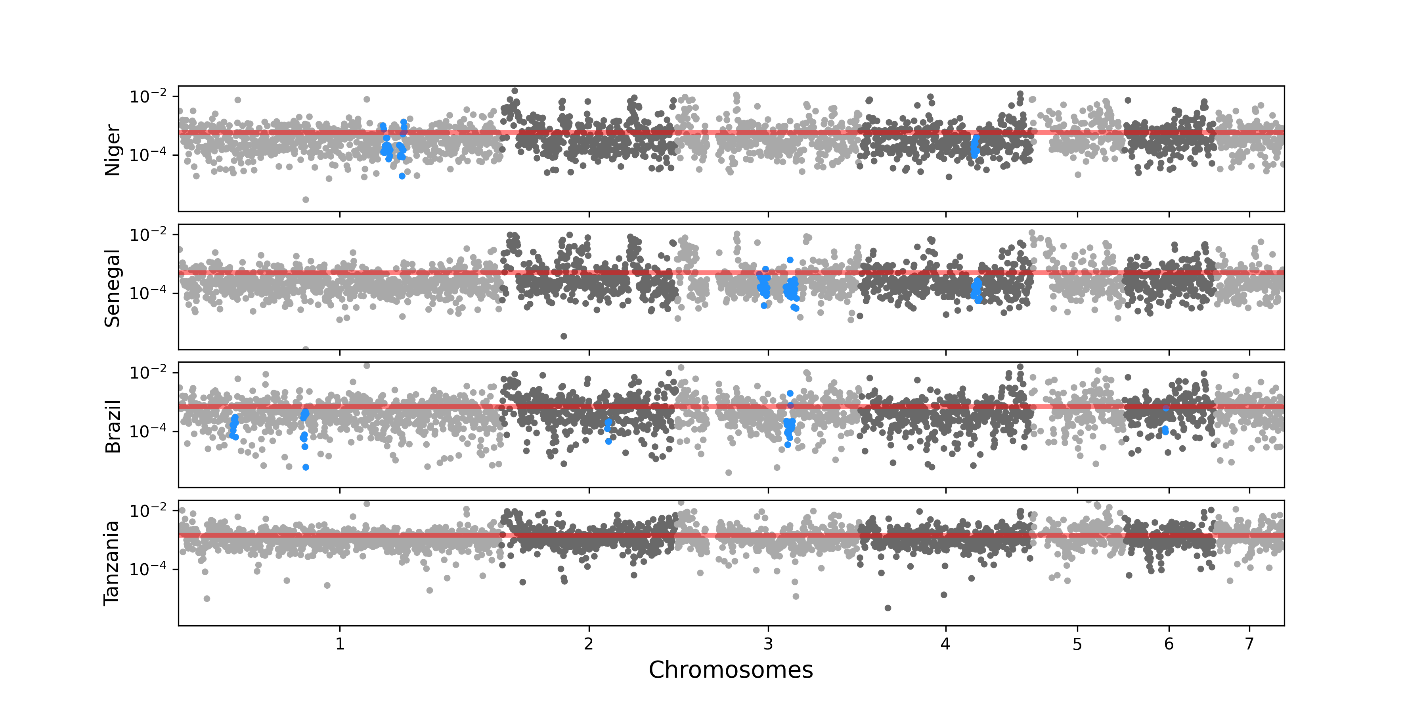
**Supplemental Figure 4. Tajma’s *D* across the genome.** π was measured across the genome in each *S. mansoni* population. Average Tajma’s *D* for each population is indicated by the red line. Tajma’s *D* in regions identified as putative regions of selection are shown in blue. Tajma’s *D* was significantly lower in putative regions of selection than expected based on genome-wide averages in Niger, Senegal, and Brazil. We did not identify any putative regions of selection in the Tanzanian population.
